# Synthesis and Antiviral Activity of Novel β-D-N4-Hydroxycytidine Ester Prodrugs as Potential Compounds for the Treatment of SARS-CoV-2 and Other Human Coronaviruses

**DOI:** 10.3390/ph17010035

**Published:** 2023-12-26

**Authors:** Elizaveta S. Darnotuk, Andrei E. Siniavin, Natal’ya S. Shastina, Sergey I. Luyksaar, Anna M. Inshakova, Natalia E. Bondareva, Sergey A. Zolotov, Nadezhda L. Lubenec, Anna B. Sheremet, Denis Y. Logunov, Nailya A. Zigangirova, Vladimir A. Gushchin, Alexander L. Gintsburg

**Affiliations:** 1 Federal State Budget Institution “National Research Centre for Epidemiology and Microbiology Named after Honorary Academician N. F. Gamaleya” of the Ministry of Health of the Russian Federation, 123098 Moscow, Russia; mslizirichi@yandex.ru (E.S.D.); inosit@yandex.ru (N.S.S.); luyksaar@gmail.com (S.I.L.); sinebryukhovaanna@mail.ru (A.M.I.); nataliia.d@mail.ru (N.E.B.); duim50@gmail.com (S.A.Z.); nlubenec@mail.ru (N.L.L.); anna-pimenova@mail.ru (A.B.S.); ldenisy@gmail.com (D.Y.L.); zigangirova@mail.ru (N.A.Z.); gintsburg@gamaleya.org (A.L.G.); 2 Institute of Fine Chemical Technologies, MIREA—Russian Technological University, 119571 Moscow, Russia; 3 Department of Molecular Neuroimmune Signaling, Shemyakin-Ovchinnikov Institute of Bioorganic Chemistry, Russian Academy of Sciences, 117997 Moscow, Russia

**Keywords:** antivirals, SARS-CoV-2, N4-hydroxycytidine, COVID-19, prodrug, ester derivatives, carboxylic acids

## Abstract

The spread of COVID-19 infection continues due to the emergence of multiple transmissible and immune-evasive variants of the SARS-CoV-2 virus. Although various vaccines have been developed and several drugs have been approved for the treatment of COVID-19, the development of new drugs to combat COVID-19 is still necessary. In this work, new 5′-O-ester derivatives of N4-hydroxycytidine based on carboxylic acids were developed and synthesized by Steglich esterification. The antiviral activity of the compounds was assessed in vitro—inhibiting the cytopathic effect of HCoV-229E, and three variants of SARS-CoV-2, on huh-7 and Vero E6 cells. Data have shown that most synthesized derivatives exhibit high activity against coronaviruses. In addition, the relationship between the chemical structure of the compounds and their antiviral effect has been established. The obtained results show that the most active compound was conjugate SN_22 based on 3-methyl phenoxyacetic acid. The results of this study indicate the potential advantage of the chemical strategies used to modify NHC as a promising avenue to be explored in vivo, which could lead to the development of drugs with improved pharmacological properties that potently inhibit SARS-CoV-2.

## 1. Introduction

The global pandemic of coronavirus in 2019 (COVID-19), caused by severe acute respiratory syndrome coronavirus 2 (SARS-CoV-2), has resulted in >770 million infections and 6.9 million deaths worldwide as of September 2023 [1]. The continuous occurrence of mutations and recombinations in the viral genome has led to the emergence of new variants of the virus. The World Health Organization (WHO) has classified various SARS-CoV-2 variants of concern (VOCs) as Alpha (B.1.1.7), Beta (B.1.351), Delta (B.1.671.2) and Omicron (B.1.1.529) [2,3]. Today, several effective therapies exist and a number of vaccines have been successfully developed to overcome the pandemic crisis. Given the current limited effectiveness of vaccines against new variants of SARS-CoV-2 with immune escape mutations, their highly pathogenic and hyper-transmissible properties, there is an urgent need to develop orally bioavailable and effective drugs with a broad spectrum of antiviral action for the prevention and treatment of COVID-19 [4].

Nucleoside analogues (NAs) play an important role in the treatment of viral respiratory diseases, especially during the current COVID-19 pandemic. Among them are remdesivir, molnupiravir, azvudine and bemnifosbuvir, which have been approved or are being investigated in clinical trials for the treatment of COVID-19 [5,6]. These drugs target a key component of the viral replication cycle—RNA-dependent RNA polymerase (RdRp).

Remdesivir (RDV, GS-5734) was the first NA drug to receive emergency-use authorization for the treatment of COVID-19 and is currently approved in more than 50 countries [7,8]. A significant limitation of the use of remdesivir is its intravenous infusion in a hospital setting only.

Molnupiravir (EIDD-2801) is an orally bioavailable 5′-isobutyryl prodrug of β-D-N4-hydroxycytidine ribonucleoside (NHC; EIDD-1931). The parent nucleoside demonstrated low oral bioavailability in animal models. It was rapidly metabolized in primate enterocytes following oral administration [9]. Molnupiravir has a high plasma level and rapid distribution in various tissues after oral administration. Molnupiravir is activated through metabolism in the body. Once inside the cell, it becomes an RNA-like component. The molecular mechanism of antiviral action lies in the ability of NHC to increase the frequency of mutations in viral RNA and impair the replication of SARS-CoV-2 in cellular and animal models of infection [10]. The main target of NHC-induced RNA mutagenesis is the viral RdRp. RdRp uses the active form of molnupiravir, β-D-N4-hydroxycytidine triphosphate, as a substrate, instead of cytidine triphosphate or uridine triphosphate. When RdRp uses RNA as a template, NHC induces the incorporation of either G or A, resulting in the formation of mutated RNA. At the end, mutations are formed in the positive-stranded viral RNA products due to the presence of NHC triphosphate, which prevent the formation of infectious and replication-capable viruses [11]. This mechanism of mutagenesis is applicable to various viral polymerases and may explain the antiviral activity of NHC against various viruses [12,13,14,15].

Transport of nucleosides and their analogues through the gastrointestinal tract is often mediated by passive diffusion or active transporters. However, the physicochemical properties of NAs are unsuitable for passive intracellular absorption in the intestine. In addition, they are not natural substrates and exhibit low affinity for nucleoside transporters. Consequently, oral absorption of nucleoside analogues is often limited [16].

To trigger antiviral effects, nucleoside analogues require intracellular phosphorylation, which is catalyzed by several host cell enzymes that convert the nucleoside into monophosphate, diphosphate, and finally into active triphosphate forms. Therefore, during the development of antiviral agents, intracellular activation by active phosphorylation and associated metabolism should be taken into account [17,18]. 

To overcome the limitations in the use of approved and developed drugs based on nucleoside analogues with anti-SARS-CoV-2 activity, the development of drugs with high oral bioavailability and high efficiency of metabolism to their triphosphate forms is required, which often leads to an increase in their antiviral activity [16,19,20].

Since the polarity of nucleosides results in their low cellular permeability and bioavailability, increasing efforts are focused on overcoming these difficulties through the development of their prodrug forms. The development of such nucleoside derivatives results in reduced cytotoxicity, improved cell penetration, improved pulmonary exposure, selectivity of action, and higher oral bioavailability compared to the parent nucleosides [21,22,23].

In a prodrug, nucleoside analogues are covalently linked to the modifying moiety through phosphodiester, ester, carbamate, amide and other types of bonds [24,25]. The sensitivity of these chemical bonds to enzymatic or chemical hydrolysis has a significant impact on the effectiveness of such prodrugs [26,27,28].

To improve the oral absorption of nucleoside analogues, the development of their prodrugs, where the hydroxyl groups of the sugar moiety are esterified with carboxylic acids or amino acids, is widely used. Prodrugs based on carboxylic acid esters or amino acids usually have significant improvements in cell membrane permeability, enzymatic stability and bioavailability [29,30].

Limitations in the chemical and biological properties of NAs against SARS-CoV-2 require the use of prodrug strategies for the development of effective drugs. The use of prodrugs can optimize the pharmacokinetic properties of the parent NAs, such as absorption, distribution, metabolism, excretion, which not only increases the effectiveness of the drug, but also reduces systemic and/or adverse tissue/organ toxicity [30].

The prodrug approach is based on the specific design of the prodrug, which allows it to cross a membrane or metabolic barrier and, having passed it, release a pharmacophore through post-barrier enzymatic or non-enzymatic processes [29,31]. Prodrugs undergo metabolic bioconversion to the active parent drug by functionally relevant and diversity-tolerant hydrolases such as peptidases, phosphatases and carboxylesterases [32,33,34].

The development and synthesis of ester derivatives is one of the most common approaches to obtaining prodrugs; for example, 5′-O-substituted NA prodrugs can often increase antiviral activity and delivery across cellular barriers, lead to changes in pharmacokinetic properties, increase plasma half-life, and site-specific drug targeting and accumulation [35]. An example of this approach is the modification of nucleoside inhibitors of HIV. The 5′-O-myristoyl and 5′-O-1,2-azidodecanoyl derivatives of lamivudine (3TC) exhibited significantly higher anti-HIV activity than the parent compound [36]. Similarly, the 5′-O-myristoyl derivative of emtricitabine (FTC) showed 10–24 times higher anti-HIV activity than the parent drug [37].

This study was devoted to the development of ester prodrugs of β-D-N4-hydroxycytidine with phenylcarboxylic acids containing various bioisosteric substitutions at the phenyl moiety and/or methylene group and studying their activity against SARS-CoV-2. We mainly performed a bioisosteric replacement of the functional group of molnupiravir (replacement of isopropyl ether with phenylcarboxylic acid esters). Bioisosterism is an approach for rational drug discovery that involves identifying molecular fragments that are interchangeable and do not result in any significant perturbation in the biological activity of the drug molecule [38,39,40]. In addition, this type of molecular editing of prodrugs with similar functional group (bioisosteres of ester derivatives) can be useful for optimizing the biological properties of molecules. This approach helps to increase pharmacological activity of the compound, selectivity, and reduce toxicity, which leads to the creation of more effective and safe drugs [41,42].

The antiviral activity prodrugs of β-D-N4-hydroxycytidine with phenylcarboxylic acids has not been studied previously, and we focused on the synthesis of such compounds and studied the effect of various modifications of the ester structure on the biological activity. Due to the different physico-chemical properties of various substituents in the phenylcarboxylic acids residues, they may have different effects on prodrug metabolism, which may lead to improved pharmacological properties [43].

The aim of this study was to develop novel β-D-N4-hydroxycytidine ester derivatives with carboxylic acids as potential antiviral agents. Analysis of the relationship between the inhibitory activity of compounds and their structure will make progress in the design of nucleoside prodrugs with high antiviral efficiency.

## 2. Results and Discussion 

### 2.1. Chemistry

**Scheme 1:**
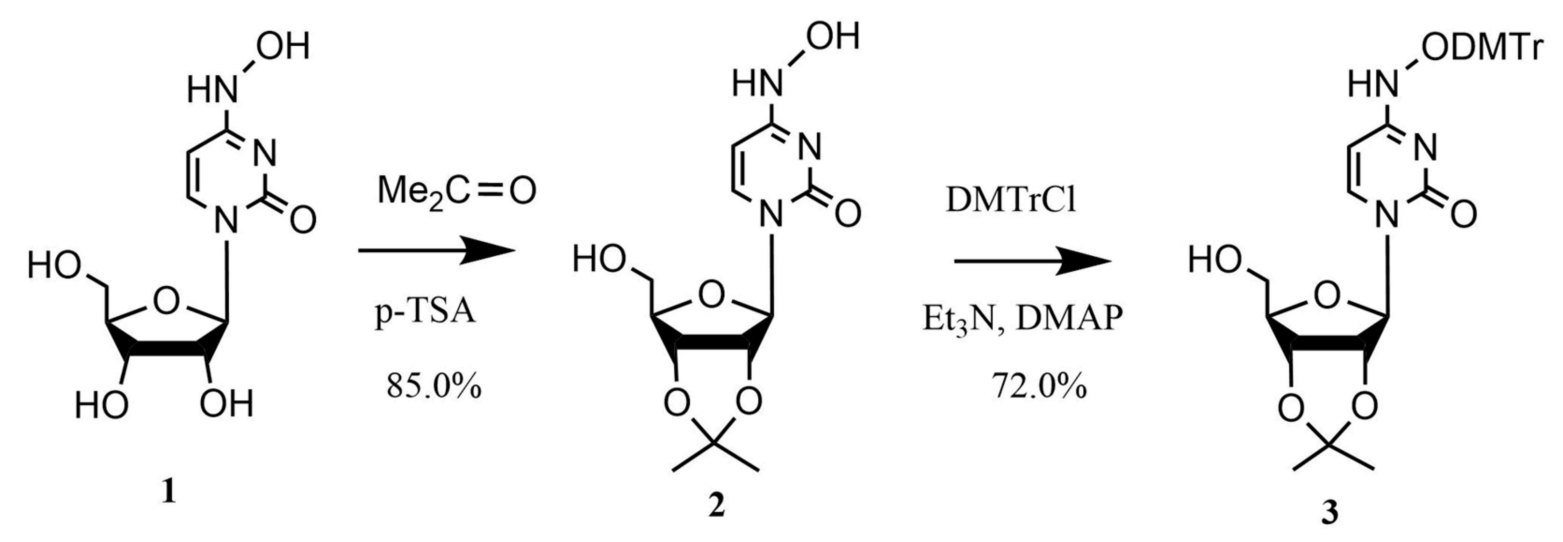
Introduction of protecting groups into β-D-N4-hydroxycytidine.

To obtain a β-D-N4-hydroxycytidine (NHC) derivative based on carboxylic acids, the vicinal 2′ and 3′ hydroxyl groups of NHC were protected using acetonide, followed by protecting of the hydroxyl group at the 4-position of N-4-hydroxycytidine with 4,4′-dimethoxytrityl chloride (DMTrCl) according to the method described in [44] (Scheme 1).

**Scheme 2:**
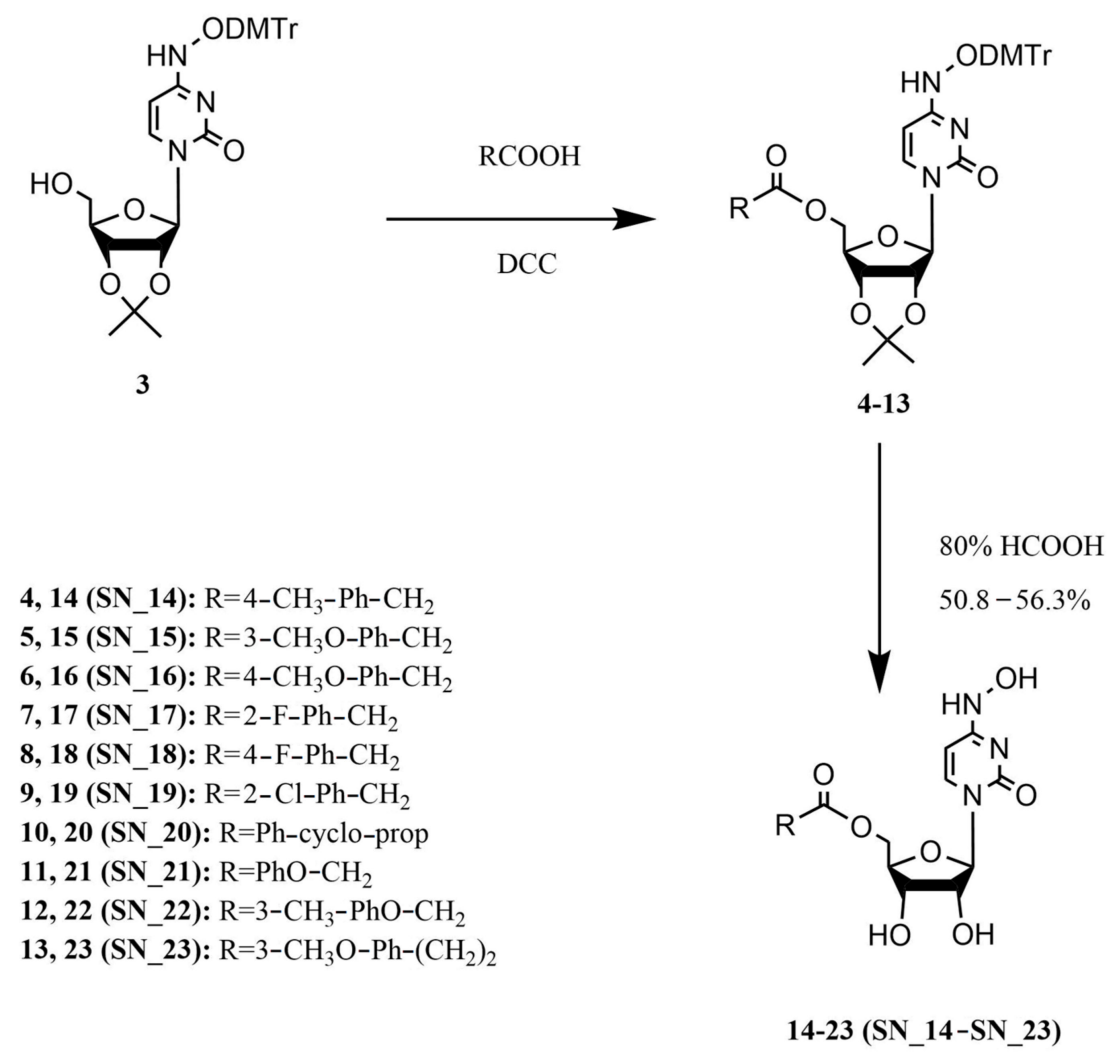
Nucleoside conjugation with carboxylic acids.

**Figure 1:**
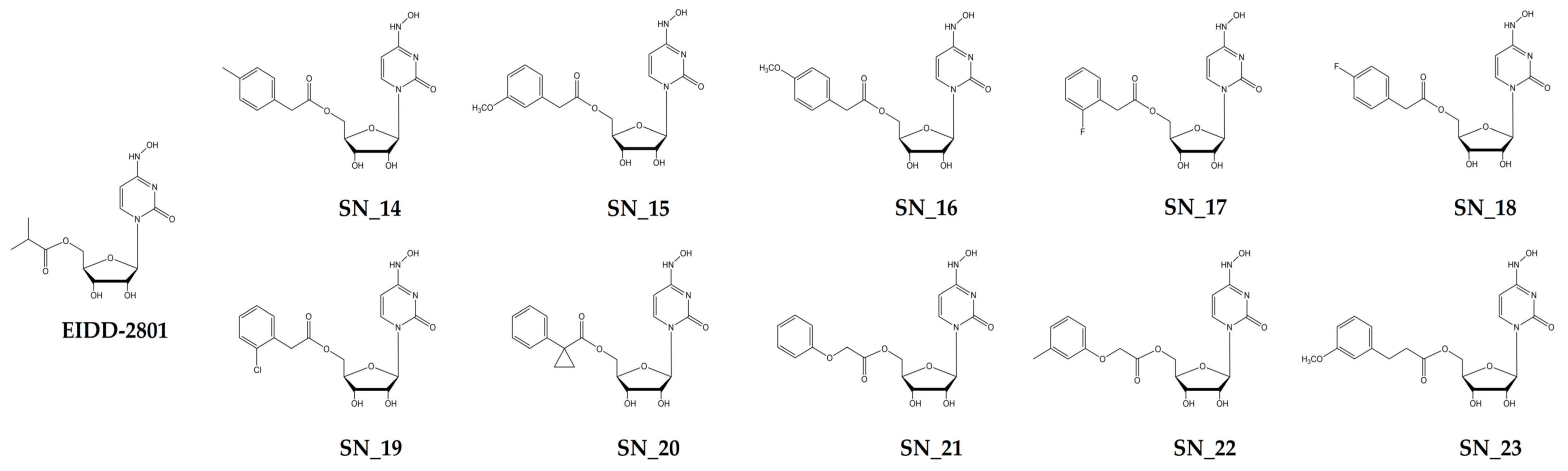
Structures of ester derivatives of β-D-N4-hydroxycytidine.

During this work, the synthesis of ester derivatives of β-D-N4-hydroxycytidine was performed by the Steglich esterification reaction of N4-dimethoxytrityloxy-2′,3′-O-isopropylidenecytidine with carboxylic acids under the action of 1,3-dicyclohexylcarbodiimide in methylene chloride at room temperature for 2 h. The purification of reaction mixtures on silica gel gave the conjugates 4–13 as crude products that were subjected to acid hydrolysis with an 80% aqueous solution of formic acid at room temperature for 20 h (Scheme 2). Compounds **14**–**23** (Figure 1) were isolated by flash chromatography on silica gel. The structure of the synthesized compounds was confirmed by NMR spectroscopy and mass spectrometry (All spectral data are available in the Supplementary Materials).

### 2.2. Antiviral Activity 

**Figure 2:**
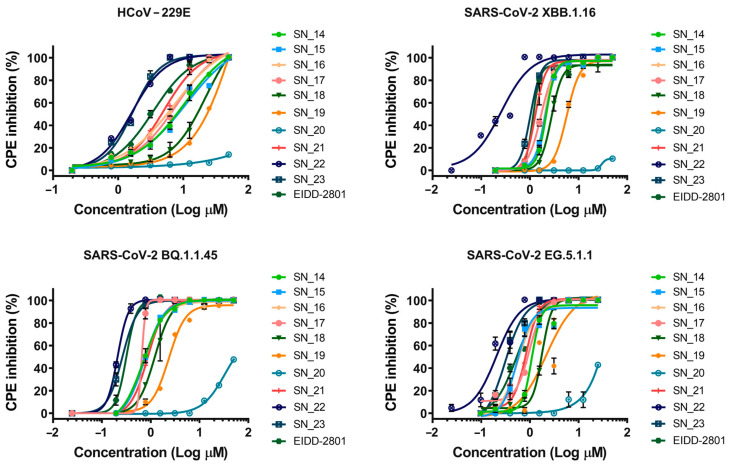
Dose-dependent inhibition of the cytopathic effect (CPE) of coronaviruses by the studied compounds. The data are presented as mean ± s.e.m. (n = 3).

Synthesized carboxylic acid ester derivatives of NHC were evaluated in vitro against various human coronavirus variants. In this study, we used human α-coronavirus of HCoV-229E and three current circulating SARS-CoV-2 variants. The results revealed interesting features of the relationship between the structure of the compounds and their antiviral effect (Table 1, Figure 2). We found that the ester derivative of NHC based on phenyl-cyclopropyl carboxylic acid (SN_20) has no antiviral effect against all tested coronavirus variants. This feature can be explained by steric hindrance in the enzymatic hydrolysis of the conjugate and its subsequent conversion into the active metabolite. These results are confirmed by previous studies where it was shown that cyclopropane carboxylic acid esters of valacyclovir have significant stability to hydrolysis (half-life >300 h) [45]. The cyclopropyl group provides hyperconjugative stabilization and has conjugative properties that make it similar to a carbon–carbon double bond in interaction with adjacent π-electron systems, which provides increased stability to hydrolysis and subsequent metabolism [46,47].

The different position of the methoxy group in the phenylacetic acid-based ester derivatives of SN_15 and SN_16 did not affect on their antiviral activity. However, conjugate SN_23 based on 3-methoxyphenylpropanoic acid ester, which is structurally similar to these two synthesized derivatives, exhibited a 2–6 times higher antiviral effect. Moreover, we found differences in the activity of fluorophenylacetic esters (SN_17 and SN_18). Substitution of phenylacetic acid with fluorine at the 2-position in the SN_17 conjugate resulted in a ≥2-fold increase in antiviral activity compared to the 4-fluoro substituted conjugate SN_18. At the same time, their antiviral effect was more pronounced compared to a similar ester derivative of 2-chlorophenylacetic acid (SN_19). The ester derivative of NHC based on 3-methyl phenoxyacetic acid (SN_22) showed higher antiviral activity compared to the conjugate based on phenoxyacetic acid (SN_21). Conjugate SN_22 showed the highest activity in inhibiting the replication of coronaviruses among all tested compounds, including EIDD-2801. In conclusion, almost all synthesized ester conjugates of NHC based on carboxylic acids showed similar or greater antiviral activity compared to EIDD-2801.

Differences in the antiviral activity of the studied ether derivatives may be explained by differences in how they are hydrolysed and metabolised. Following oral administration, these NHC prodrugs undergo hydrolysis and subsequent metabolism, as previously shown for similar prodrugs [19,48,49]. In the derivatives of phenylpropionic acid and phenoxyacetic acid (SN_21-SN_23), the distance of the phenyl radical from the carbonyl of the ester group apparently leads to an easier nucleophilic attack of the OH-serine in the active site of the carboxylesterase on the ester group with the formation of an enzyme–substrate complex and subsequent hydrolysis, reduces the hydrolysis time and increases metabolism.

For phenylacetic acid derivatives (SN_14-SN_19), the accessibility of the active center of carboxylesterase for the formation of an enzyme–substrate complex is difficult due to the proximity of the phenyl residue to the carbonyl of the ester bond. This slows down the hydrolysis of these compounds under the action of the enzyme.

The electronic effects of substituents on the phenyl residue do not have a noticeable effect on the metabolism of ester derivatives, which is consistent with previous data showing a significant influence on the rate of hydrolysis of such prodrugs by steric rather than electronic factors, which leads to pharmacological differences between them [50]. 

It is known that the sensitivity to hydrolysis increases slightly with a decrease in the electron density in the benzene ring of the ester substrate [51]. For compounds SN_14-SN_18, differences in metabolic activity may be associated with a decrease in the electron density in the aromatic ring of the CH_3_, CH_3_O, F substituents. The reduced antiviral activity of the SN_19 derivative containing a chlorophenylacetic acid residue is apparently due to the fact that in the case of aromatic esters the rate of hydrolysis decreases with increasing van der Waals radius of the substituent in the benzene ring.

## 3. Conclusions

In this work, a series of ester derivatives of NHC based on carboxylic acids were developed and tested, and their relationship between the carboxylic acid used and antiviral activity was established. Most of the synthesized compounds are equally effective or have greater activity than EIDD-2801. Conjugate SN_20 based on 1-phenyl-1-cyclopropylcarboxylic acid showed the lowest activity. Ester derivative SN_22 based on 3-methylphenoxyacetic acid had the most powerful antiviral effect. Based on our results, it is shown that the nucleoside modification approach we developed may have the potential to discover new antiviral drugs for the treatment of COVID-19 and other human coronavirus infections. The SN_22 conjugate, which showed the greatest antiviral effect, can be considered as a lead compound for the creation of antiviral drugs in the future. Future studies of the hydrolysis, biotransformation and metabolism of NHC ether derivatives will allow us to select the most promising compounds for in vivo studies.

## 4. Materials and Methods

### 4.1. Chemistry

^1^H-NMR spectra were recorded on a Bruker DPX-300 pulsed NMR spectrometer (Bruker BioSpin, Rheinstetten, Germany) with an operating frequency of 300 MHz in deuterated solvents. The chemical shifts of protons are given relative to the internal standard, tetramethylsilane (0.0 ppm). Mass spectrometry was carried out on a Bruker autoflex speed MALDI mass spectrometer (Bruker Daltonics, Bremen, Germany), the following compounds were used as matrices: 3-indoleacrylic acid (IAA), α-cyano-4-hydroxycinnamic acid (CHCA), sinapic acid (SIN).

We used hexane, chloroform, triethylamine, methanol and methylene chloride. Chloroform was purified by distillation over calcium chloride, methylene chloride was distilled over phosphorus pentoxide, triethylamine was purified by distillation over potassium hydroxide and then boiling for 3–5 h over calcium hydride followed by distillation.

The following reagents were used in the work: β-D-N4-hydroxycytidine (NHC), phenylcarboxylic acids (Sigma-Aldrich, St. Louis, MO, USA), DCC (Lancaster, Lancashire, UK), DMAP (Merck, Darmstadt, Germany), as well as sulfuric acid, formic acid, sodium sulfate, sodium bicarbonate (Chimmed, Moscow, Russia).

Thin-layer chromatography (TLC) was carried out on Sorbfil plates (Sorbpolimer, Krasnodar, Russia) in the following solvent system:

Chloroform: methanol: aqueous ammonia (7.5:2.5:0.2)

The detection of spots on chromatograms was carried out in UV light, by treatment with 10% H_2_SO_4_ in MeOH, followed by heating at 200 °C.

Flash chromatography was performed on Silica gel (0.040–0.063 mm) (Merck, Darmstadt, Germany).

#### 4.1.1. Synthesis of 5′-O-(4-Methylphenyl)acetyl-N4-hydroxycytidine (**14**, SN_14)

A solution of 150 mg (0.249 mmol) of substituted nucleoside (**3**), 56 mg (0.374 mmol) (4-methylphenyl)acetic acid and 115 mg (0.560 mmol) DCC in methylene chloride (15 mL) was stirred at room temperature for 2 h. The reaction the mass was evaporated, the residue was prepurified on silica gel in the system chloroform:hexane:triethylamine (7:3:0.25). Intermediate product (**4**), without individual isolation, was exposed to 80% aqueous formic acid (5 mL), stirred at room temperature for 20 h, evaporated and purified by flash chromatography on silica gel in the system chloroform:methanol (5% methanol).

14, SN_14: yield 54.8 mg (56.3%, amorphous), R_f_ 0.42.

^1^H-NMR (DMSO-*d*_6_, δ, ppm): 2.27 (s. 3 H, CH_3_-Ph), 3.66 (s, 2H, CH_2_-CO), 3.86–4.18 (2m, 4H, 5′-CH_2_, 2OH Rib), 4.19–4.26 (m, 1H, 4′-CH), 5.11–5.31 (2m, 2H, 2′ и 3′-CH), 5.53 (d, J = 8.1 Hz, 1H, 1′-CH), 5.70 (d, J = 5.2 Hz, 1H, H-5 Cyt), 6.67 (d, J = 8.2 Hz, 1H, H-6 Cyt), 7.08–7.18 (2m, 4H, Ph), 9.47 (s, 1H, NHOH).

MS (MALDI-TOF): *m*/*z* calcd. fo C_18_H_21_N_3_O_7_ 391.4; founded 414.46 [M + Na^+^].

#### 4.1.2. Synthesis of 5′-O-(3-Methoxyphenyl)acetyl-N4-hydroxycytidine (**15**, SN_15)

Prepared analogously to compound (**14**) from 150 mg (0.249 mmol) of substituted nucleoside (**3**), 67 mg (0.374 mmol) (3-methoxyphenyl)acetic acid and 115 mg (0.560 mmol) DCC in methylene chloride (30 mL), product (**15**) was purified by flash chromatography on silica gel in a mixture of chloroform:methanol (5% methanol).

15, SN_15: yield 49.9 mg (50.8%, amorphous), R_f_ 0.44.

^1^H-NMR (DMSO-*d*_6_, δ, ppm): 3.70 (s, 2H, CH_2_-CO), 3.73 (s, 3H, CH_3_O-Ph), 3.87–4.16 (2m, 4H, 5′-CH_2_, 2OH Rib), 4.19–4.25 (m, 1H, 4′-CH), 5.15–5.40 (2m, 2H, 2′ и 3′-CH), 5.54 (d, J = 8.2 Hz, 1H, 1′-CH), 5.70 (d, J = 5.0 Hz, 1H, H-5 Cyt), 6.68 (d, J = 8.2 Hz, 1H, H-6 Cyt), 6.78–7.27 (2m, 4H, Ph), 9.49 (s, 1H, NHOH).

MS (MALDI-TOF): *m*/*z* calcd. fo C_18_H_21_N_3_O_8_ 407.38; founded 430.37 [M + Na^+^].

#### 4.1.3. Synthesis of 5′-O-(4-Methoxyphenyl)acetyl-N4-hydroxycytidine (**16**, SN_16)

Prepared analogously to compound (**14**) from 150 mg (0.249 mmol) of substituted nucleoside (**3**), 62 mg (0.374 mmol) (4-methoxyphenyl)acetic acid and 115 mg (0.560 mmol) DCC in methylene chloride (30 mL), product (**16**) was purified by flash chromatography on silica gel in a mixture of chloroform:methanol (5% methanol).

16, SN_16: yield 55 mg (54.2%, amorphous), R_f_ 0.41.

^1^H-NMR (DMSO-*d*_6_, δ, ppm): 3.64 (s, 2H, CH_2_-CO), 3.73 (s, 3H, CH_3_O-Ph), 3.84–4.17 (2m, 4H, 5′-CH2, 2OH Rib), 4.19–4.25 (m, 1H, 4′-CH), 5.13–5.32 (2m, 2H, 2′ и 3′-CH), 5.54 (d, J = 8.2 Hz, 1H, 1′-CH), 5.70 (d, J = 5.2 Hz, 1H, H-5 Cyt), 6.66 (d, J = 8.2 Hz, 1H, H-6 Cyt), 6.84–7.25 (2m, 4H, Ph), 9.47 (s, 1H, NHOH).

MS (MALDI-TOF): *m*/*z* calcd. fo C_18_H_21_N_3_O_8_ 407.38; founded 430.38 [M + 3Na^+^].

#### 4.1.4. Synthesis of 5′-O-(2-Fluorophenyl)acetyl-N4-hydroxycytidine (**17**, SN_17)

Prepared analogously to compound (**14**) from 150 mg (0.249 mmol) of substituted nucleoside (**3**), 58 mg (0.374 mmol) (2-fluorophenyl)acetic acid and 115 mg (0.560 mmol) DCC in methylene chloride (30 mL), product (**17**) was purified by flash chromatography on silica gel in a solvent mixture of chloroform:methanol (5% methanol).

17, SN_17: yield 52 mg (53.0%, amorphous), R_f_ 0.42.

^1^H-NMR (DMSO-*d*_6_, δ, ppm): 3.79 (s, 2H, CH_2_-CO), 3.83–4.12 (2m, 4H, 5′-CH2, 2OH Rib), 4.21–4.27 (m, 1H, 4′-CH), 5.17–5.38 (2m, 2H, 2′ и 3′-CH), 5.56 (d, J = 9.8 Hz, 1H, 1′-CH), 5.70 (d, J = 5.0 Hz, 1H, H-5 Cyt), 6.67 (d, J = 8.2 Hz, 1H, H-6 Cyt), 7.15–7.42 (2m, 4H, Ph), 9.56 (s, 1H, NHOH).

MS (MALDI-TOF): *m*/*z* calcd. fo C_17_H_18_FN_3_O_7_ 395.34; founded 418.35 [M + Na^+^].

#### 4.1.5. Synthesis of 5′-O-(4-Fluorophenyl)acetyl-N4-hydroxycytidine (**18**, SN_18)

Prepared analogously to compound (**14**) from 150 mg (0.249 mmol) of substituted nucleoside (**3**), 58 mg (0.374 mmol) (4-fluorophenyl)acetic acid and 115 mg (0.560 mmol) DCC in methylene chloride (30 mL), product (**18**) was purified by flash chromatography on silica gel in a solvent mixture of chloroform:methanol (5% methanol).

18, SN_18: yield 54.6 mg (55.4%, amorphous), R_f_ 0.42.

^1^H-NMR (DMSO-*d*_6_, δ, ppm): 3.73 (s, 2H, CH_2_-CO), 3.86–4.20 (2m, 4H, 5′-CH2, 2OH Rib), 4.21–4.27 (m, 1H, 4′-CH), 5.14–5.26 (2m, 2H, 2′ и 3′-CH), 5.56 (d, J = 8.2 Hz, 1H, 1′-CH), 5.69 (d, J = 5.1 Hz, 1H, H-5 Cyt), 6.69 (d, J = 8.2 Hz, 1H, H-6 Cyt), 7.08–7.37 (2m, 4H, Ph), 9.46 (s, 1H, NHOH).

MS (MALDI-TOF): *m*/*z* calcd. fo C_17_H_18_FN_3_O_7_ 395.34; founded 418.48 [M + Na^+^].

#### 4.1.6. Synthesis of 5′-O-(2-Chlorophenyl)acetyl-N4-hydroxycytidine (**19**, SN_19)

Prepared analogously to compound (**14**) from 150 mg (0.249 mmol) of substituted nucleoside (**3**), 63 mg (0.374 mmol) (2-chlorophenyl)acetic acid and 115 mg (0.560 mmol) DCC in methylene chloride (30 mL), product (**19**) was purified by flash chromatography on silica gel in a solvent mixture of chloroform:methanol (5% methanol).

19, SN_19: yield 50 mg (52.6%, amorphous), R_f_ 0.43.

^1^H-NMR (DMSO-*d*_6_, δ, ppm): 3.87 (s, 2H, CH_2_-CO), 3.90–4.21 (2m, 4H, 5′-CH2, 2OH Rib), 4.21–4.24 (m, 1H, 4′-CH), 5.12–5.32 (2m, 2H, 2′ и 3′-CH), 5.57 (d, J = 8.1 Hz, 1H, 1′-CH), 5.70 (d, J = 5.8 Hz, 1H, H-5 Cyt), 6.65 (d, J = 8.2 Hz, 1H, H-6 Cyt), 7.25–7.51 (2m, 4H, Ph), 9.46 (s, 1H, NHOH).

MS (MALDI-TOF): *m*/*z* calcd. fo C_17_H_18_ClN_3_O_7_ 411.80; founded 434.29 [M + Na^+^].

#### 4.1.7. Synthesis of 5′-O-(1-Phenylcyclopropanoyl-1-carbonyl)-N4-hydroxycytidine (**20**, SN_20)

Was prepared analogously to compound (**14**) from 150 mg (0.249 mmol) of substituted nucleoside (**3**), 61 mg (0.374 mmol) 1-phenylcyclopropanoic acid and 115 mg (0.560 mmol) DCC in methylene chloride (30 mL), product (**20**) was purified by flash chromatography on silica gel in a solvent mixture of chloroform:methanol (5% methanol).

20, SN_20: yield 54.6 mg (52.5%, amorphous), R_f_ 0.44.

^1^H-NMR (DMSO-*d*_6_, δ, ppm): 1.06, 1.50 (2t, J = 7.4 Hz, 4H, 2CH_2_ cyclo prop), 3.72–4.13 (2m, 4H, 5′-CH2, 2OH Rib), 4.13–4.21 (m, 1H, 4′-CH), 5.04–5.20 (2m, 2H, 2′ и 3′-CH), 5.52 (d, J = 8.2 Hz, 1H, 1′-CH), 5.62 (d, J = 6.5 Hz, 1H, H-5 Cyt), 6.21 (d, J = 8.2 Гц, 1H, H-6 Cyt), 7.19–7.39 (m, 5H, Ph), 9.44 (s, 1H, NHOH).

MS (MALDI-TOF): *m*/*z* calcd. fo C_19_H_21_N_3_O_7_ 403.39; founded 426.45 [M + Na^+^].

#### 4.1.8. Synthesis of 5′-O-Phenoxyacetyl-N4-hydroxycytidine (**21**, SN_21)

Was prepared similarly to compound (**14**) from 150 mg (0.249 mmol) of substituted nucleoside (**3**), 56 mg (0.374 mmol) phenoxyacetic acid and 115 mg (0.560 mmol) DCC in methylene chloride (30 mL), product (**21**) was purified by flash chromatography on silica gel in a mixture of chloroform:methanol (5% methanol).

21, SN_21: yield 53.4 mg (54.6%, amorphous), R_f_ 0.46.

^1^H-NMR (DMSO-*d*_6_, δ, ppm): 3.85–4.15 (2m, 4H, 5′-CH2, 2OH Rib), 4.22–4.34 (m, 1H, 4′-CH), 4.80 (s, 2H, CH_2_-CO), 5.26–5.42 (2m, 2H, 2′ и 3′-CH), 5.53 (d, J = 9.1 Hz, 1H, 1′-CH), 5.72 (d, J = 5.8 Hz, 1H, H-5 Cyt), 6.72 (d, J = 9.0 Hz, 1H, H-6 Cyt), 6.60–7.22 (2m, 4H, Ph), 9.58 (s, 1H, NHOH).

MS (MALDI-TOF): *m*/*z* calcd. fo C_17_H_19_N_3_O_8_ 393.35; founded 416.46 [M + Na^+^].

#### 4.1.9. Synthesis of 5′-O-(3-Methylphenoxy)acetyl-N4-hydroxycytidine (**22**, SN_22)

Prepared analogously to compound (**14**) from 150 mg (0.249 mmol) of substituted nucleoside (**3**), 62 mg (0.374 mmol) (3-methylphenoxy)acetic acid and 115 mg (0.560 mmol) DCC in methylene chloride (30 mL), product (**22**) was purified by flash chromatography on silica gel in a mixture of chloroform:methanol (5% methanol).

22, SN_22: yield 56 mg (55.1%, amorphous), R_f_ 0.40.

^1^H-NMR (DMSO-*d*_6_, δ, ppm): 2.36 (s, 3H, CH_3_-Ph), 3.89–4.17 (2m, 4H, 5′-CH2, 2OH Rib), 4.20–4.33 (m, 1H, 4′-CH), 4.80 (s, 2H, CH_2_), 5.24–5.41 (2m, 2H, 2′ и 3′-CH), 5.53 (d, J = 9.2 Hz, 1H, 1′-CH), 5.72 (d, J = 5.7 Hz, 1H, H-5 Cyt), 6.72 (d, J = 8.1 Hz, 1H, H-6 Cyt), 6.58–7.20 (2m, 4H, Ph), 9.58 (s, 1H, NHOH).

MS (MALDI-TOF): *m*/*z* calcd. fo C_18_H_21_N_3_O_8_ 403.38; founded 430.40 [M + Na^+^].

#### 4.1.10. Synthesis of 5′-O-3-(3-Methoxyphenyl)propanoyl-N4-hydroxycytidine (**23**, SN_23)

Was prepared analogously to compound (**14**) from 150 mg (0.249 mmol) of substituted nucleoside (**3**), 67 mg (0.374 mmol) 3-methoxyphenylpropanoic acid and 115 mg (0.560 mmol) DCC in methylene chloride (30 mL), product (**23**) was purified by flash chromatography on silica gel in a mixture of chloroform:methanol (5% methanol).

23, SN_23: yield 51 mg (51.5%, amorphous), R_f_ 0.41.

^1^H-NMR (DMSO-*d*_6_, δ, ppm): 2.66 (t, J = 7.4 Hz, 2H, CH_2_-CO), 2.82 (t, J = 7.4 Hz, 2H, CH_2_-Ph), 3.72 (s, 3H, CH_3_O-Ph), 3.85–4.17 (m, 4H, 5′-CH_2_, 2OH Rib), 4.17–4.25 (m, 1H, 4′-CH), 5.18–5.40 (2m, 2H, 2′ и 3′-CH), 5.56 (d, J = 8.2 Hz, 1H, 1′-CH), 5.71 (d, J = 5.5 Hz, 1H, H-5 Cyt), 6.75 (d, J = 4.7 Hz, 1H, H-6 Cyt), 6.78–7.22 (2m, 4H, Ph), 9.57 (s, 1H, NHOH).

MS (MALDI-TOF): *m*/*z* calcd. fo C_19_H_23_N_3_O_8_ 421.41; founded 444.50 [M + Na^+^].

### 4.2. Antiviral Assay

The antiviral activity of the compounds was studied as described previously [52]. Briefly, Vero E6 cells (ATCC, CRL-1586) or huh-7 cells (JCRB cell bank; JCRB0403) were seeded in 96-well plates the day before the experiment. Then, serial two-fold dilutions of the compounds were prepared and added to the cells monolayer. Cells were infected with SARS-CoV-2 or 229E at 100 TCID_50_ (50% tissue culture infective dose). The plates were incubated for 72–96 h at 35–37 °C and 5% CO_2_. Inhibition of the virus-induced cytopathic effect (CPE) by the compounds was assessed using the MTT method. The 50% inhibitory concentration (IC_50_) was determined by a regression analysis using GraphPad Prism 9 (GraphPad Software Inc., La Jolla, CA, USA). The following strains of coronaviruses from a laboratory collection of viruses were used in the experiment: SARS-CoV-2 BQ.1.1.45 (EPI_ISL_17480987), XBB.1.16 (EPI_ISL_17474859), EG.5.1.1 (EPI_ISL_18543696) and HCoV-229E/ASi_ESH/Russia/Moscow/2021 (GenBank accession OR909070). All viral procedures were performed in a biosafety level 3 (BSL-3) laboratory.

## Figures and Tables

**Table 1:** Cytotoxicity and antiviral activity of ester derivatives of NHC against hCoV-229E and SARS-CoV-2.

Compound, R	CC_50_, µM (Vero E6 and huh-7)	HCoV-229E (IC_50_, µM)	SARS-CoV-2 (IC_50_, µM)			Selectivity Index (SI) for SARS-CoV-2
			XBB.1.16	BQ.1.1.45	EG.5.1.1	CC_50_/IC_50_ (Mean)
SN_14 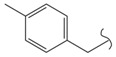	≥50	10.54	2.16	0.70	1.09	38
SN_15 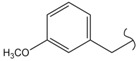		11.09	2.09	0.74	0.53	45
SN_16 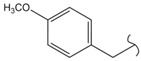		7.29	2.02	0.71	0.56	46
SN_17 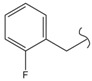		7.86	1.69	0.65	0.98	45
SN_18 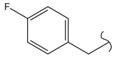		20.97	2.76	1.21	1.78	26
SN_19 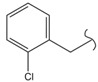		23.8	5.71	2.34	2.16	15
SN_20 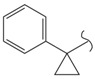		>100	>100	≥50	>100	-
SN_21 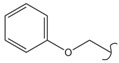		5.13	1.28	0.86	0.83	51
SN_22 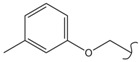		1.62	0.25	0.21	0.20	227
SN_23 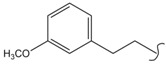		1.68	1.02	0.26	0.30	95
EIDD-2801 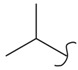		3.44	1.51	0.31	0.53	64

## Data Availability

Data are contained within the article.

## Supplementary Materials

The following supporting information can be downloaded at: https://www.mdpi.com/article/10.3390/ph17010035/s1, Spectral characterizations (MS (MALDI-TOF) and ^1^H NMR) and scanned charts of compounds.
