# Supplementary material for: Synthesis and Antiviral Activity of Novel β-D-N4-Hydroxycytidine Ester Prodrugs as Potential Compounds for the Treatment of SARS-CoV-2 and Other Human Coronaviruses
Source: Pharmaceuticals (Basel). 2023 Dec 26;17(1):35. doi: 10.3390/ph17010035 (PMC10821229; doi:10.3390/ph17010035)

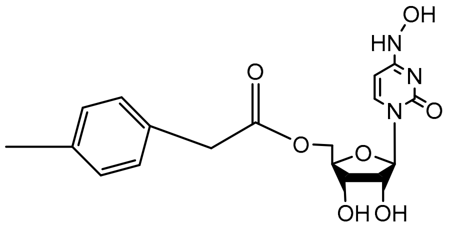

Molecular Weight: 391,38

5'-O-(4-methylphenyl)acetyl-N4-hydroxycytidine (14, SN_14)

α-Cyano-4-hydroxycinnamic acid, CHCA

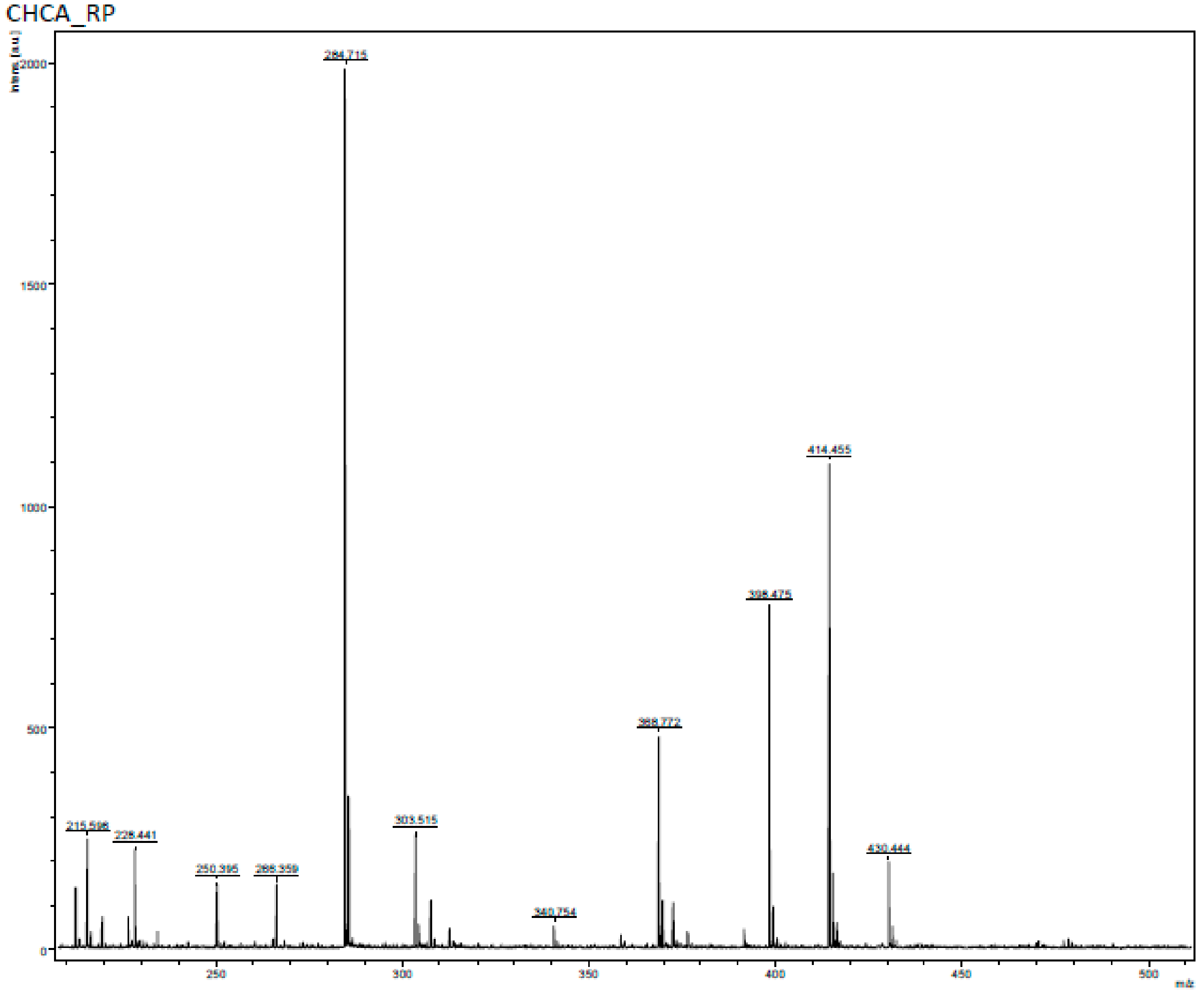

MS (MALDI-TOF): m/z calcd. for $C_{18}H_{21}N_3O_7$ 391.38; founded 414.46 [M+Na^+].

^{1}H NMR of compound **SN_14**

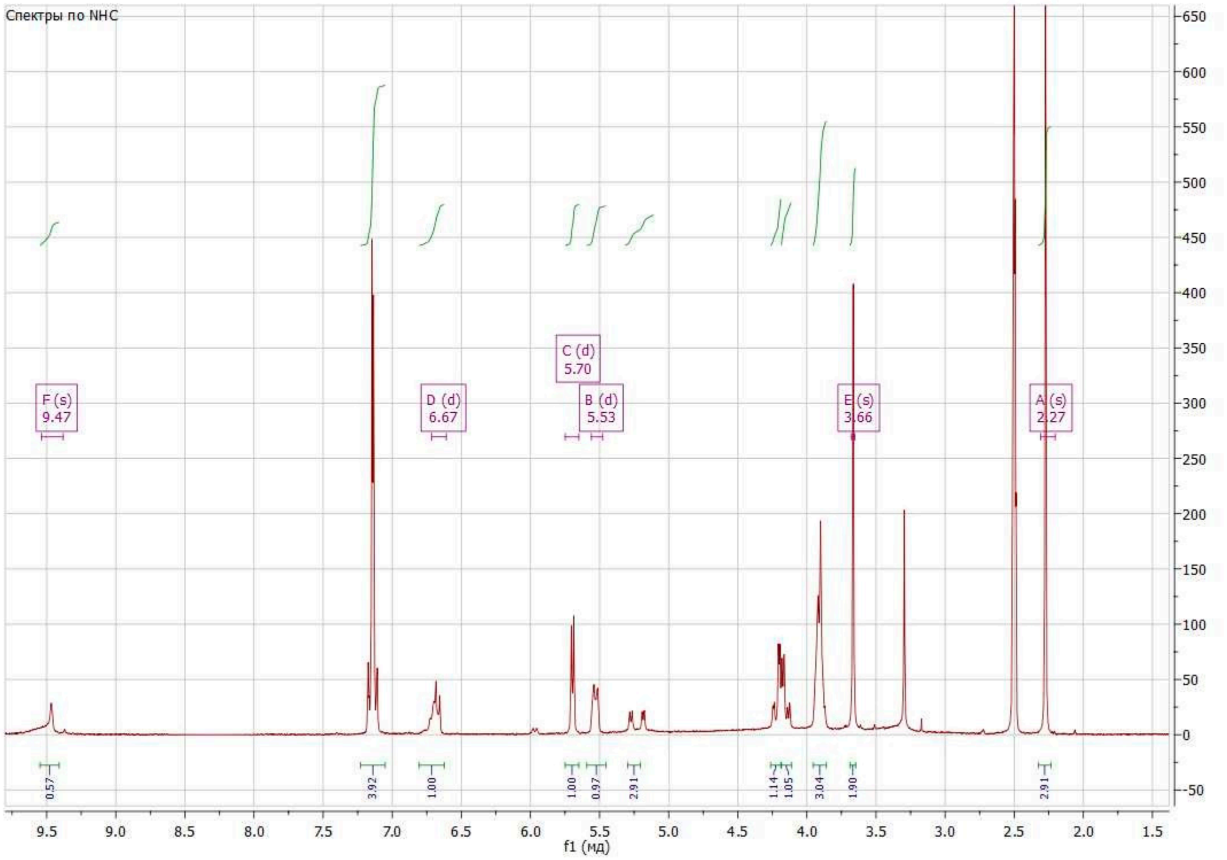

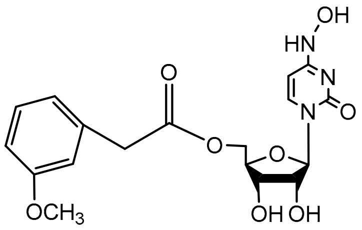

Molecular Weight: 407,38

5'-O-(3-methoxyphenyl)acetyl-N4-hydroxycytidine (15, SN_15)

3-Indoleacrylic acid, IAA

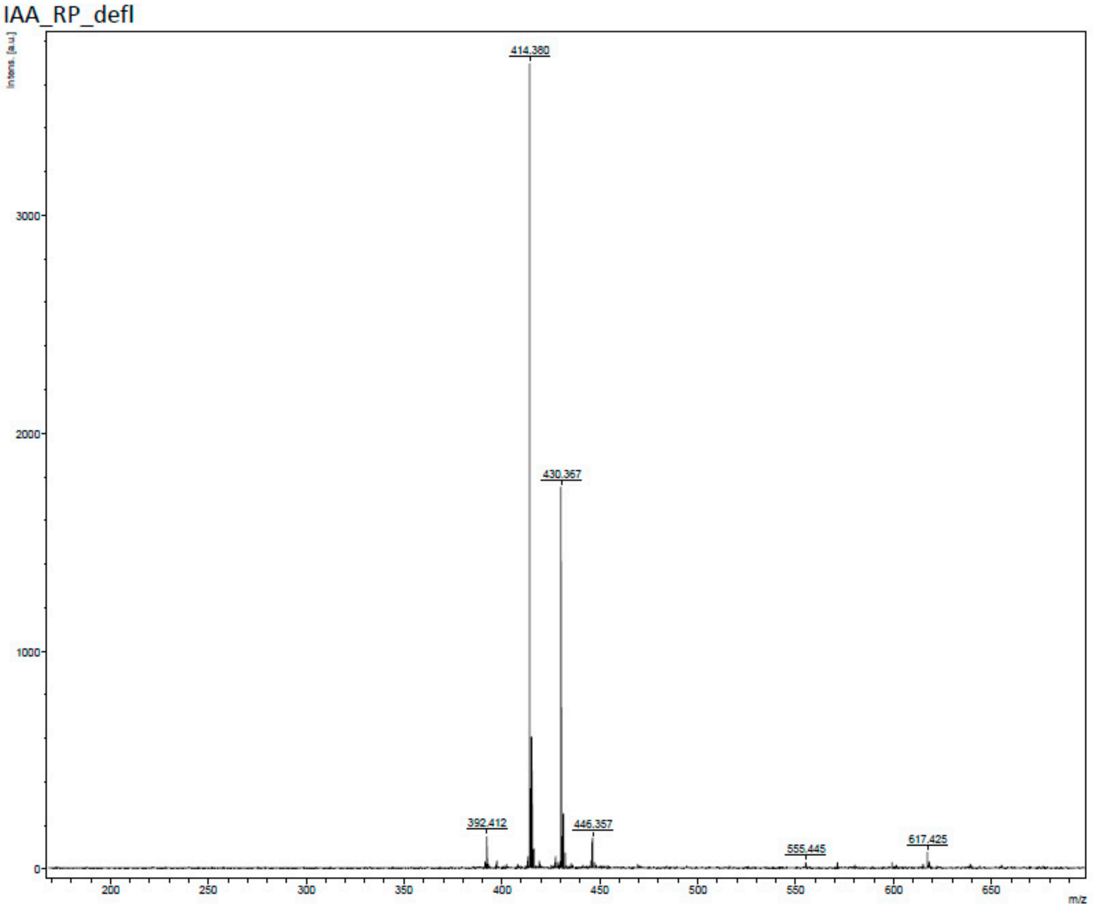

MS (MALDI-TOF): m/z calcd. for $C_{18}H_{21}N_3O_8$ 407.38; founded 430.37 [M+Na$^+$].

^{1}H NMR of compound **SN_15**

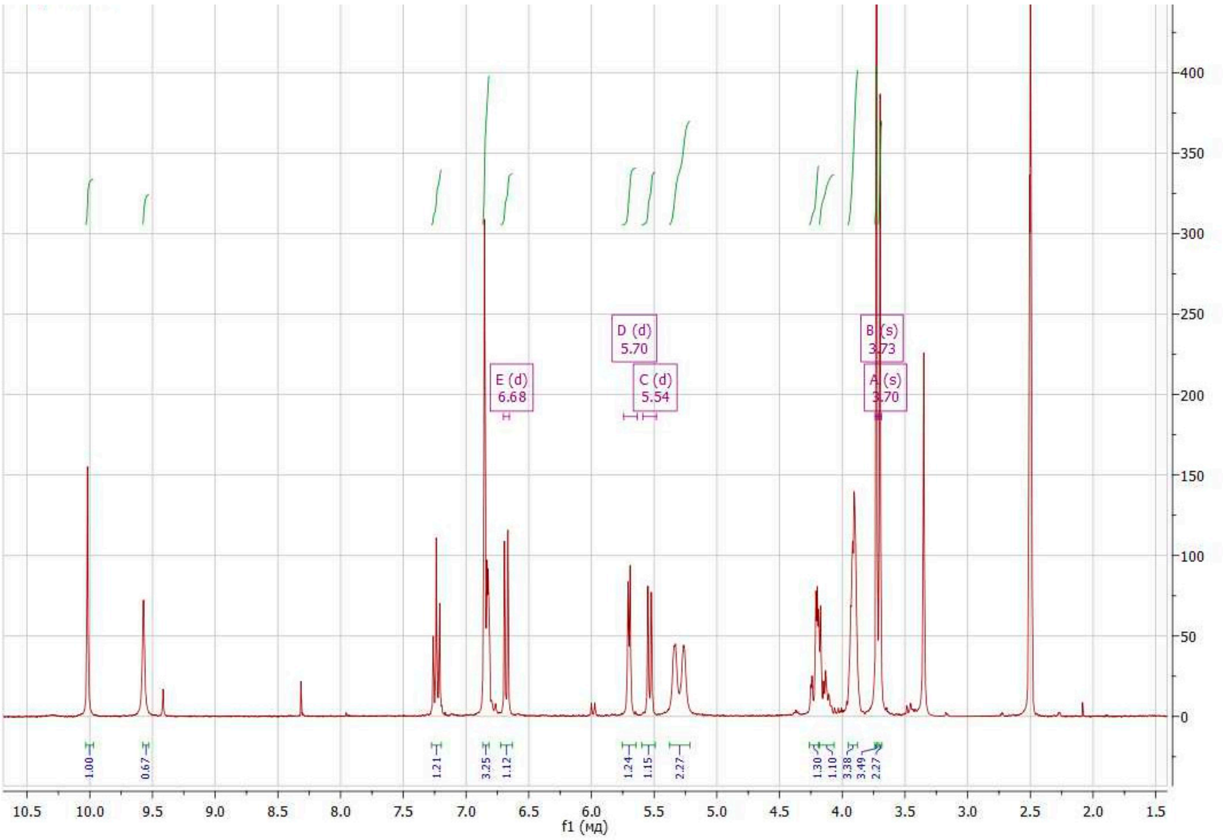

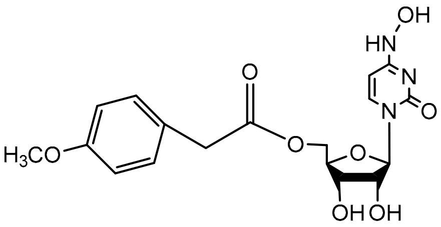

Molecular Weight: 407,38

5'-O-(4-methoxyphenyl)acetyl-N4-hydroxycytidine (16, SN_16)

3-Indoleacrylic acid, IAA

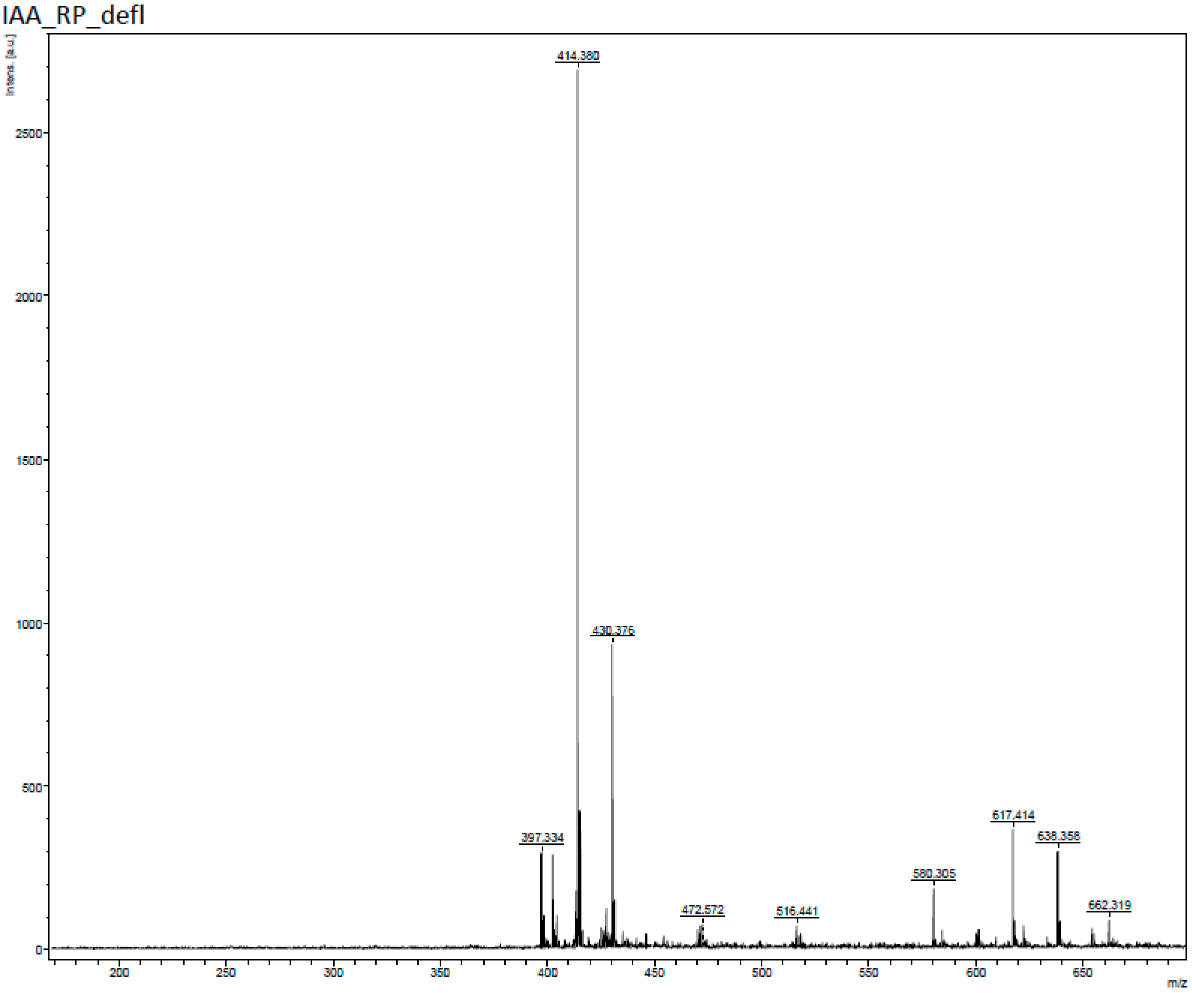

MS (MALDI-TOF): m/z calcd. for $C_{18}H_{21}N_3O_8$ 407.38; founded 430.38 [M+Na+].

^{1}H NMR of compound **SN_16**

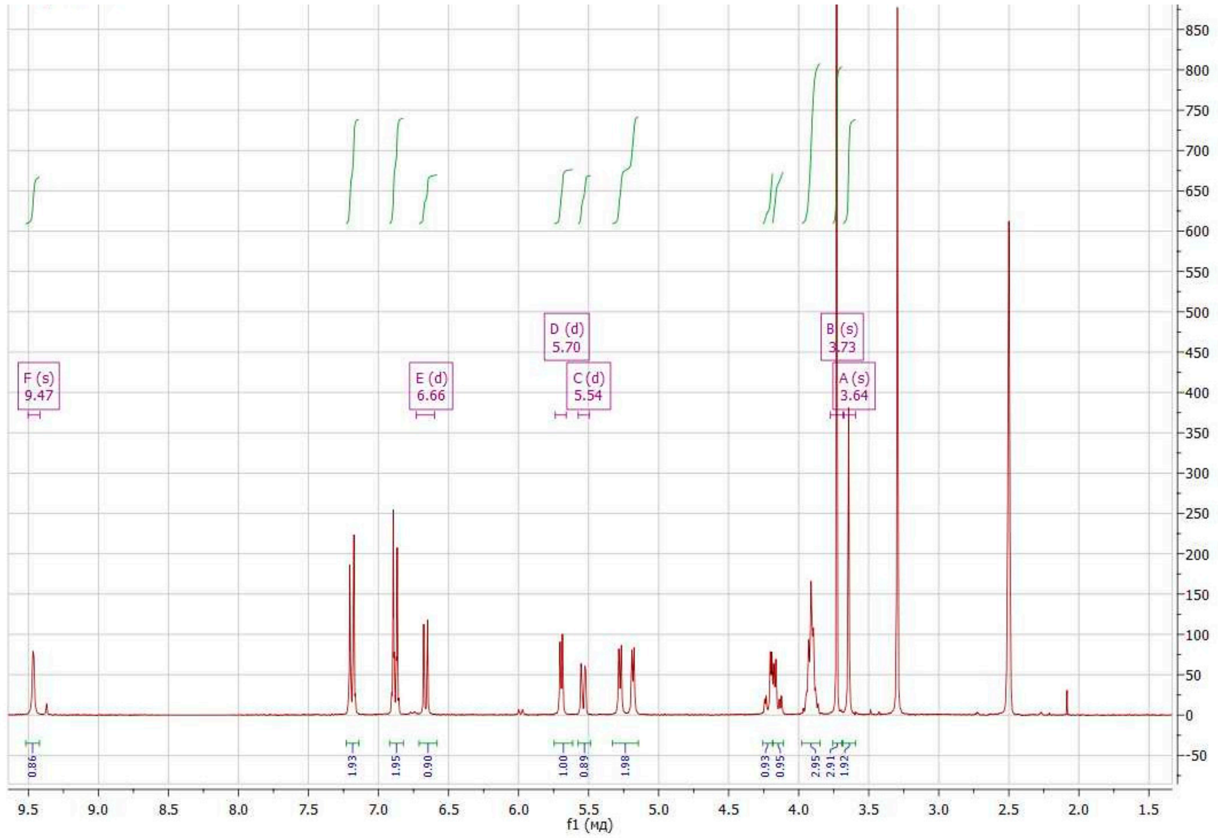

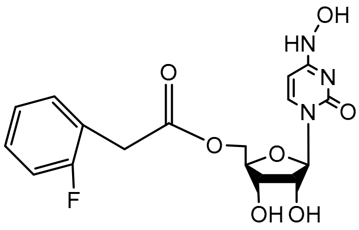

Molecular Weight: 395,34

5'-O-(2-fluorophenyl)acetyl-N4-hydroxycytidine (17, SN_17)

3-Indoleacrylic acid, IAA

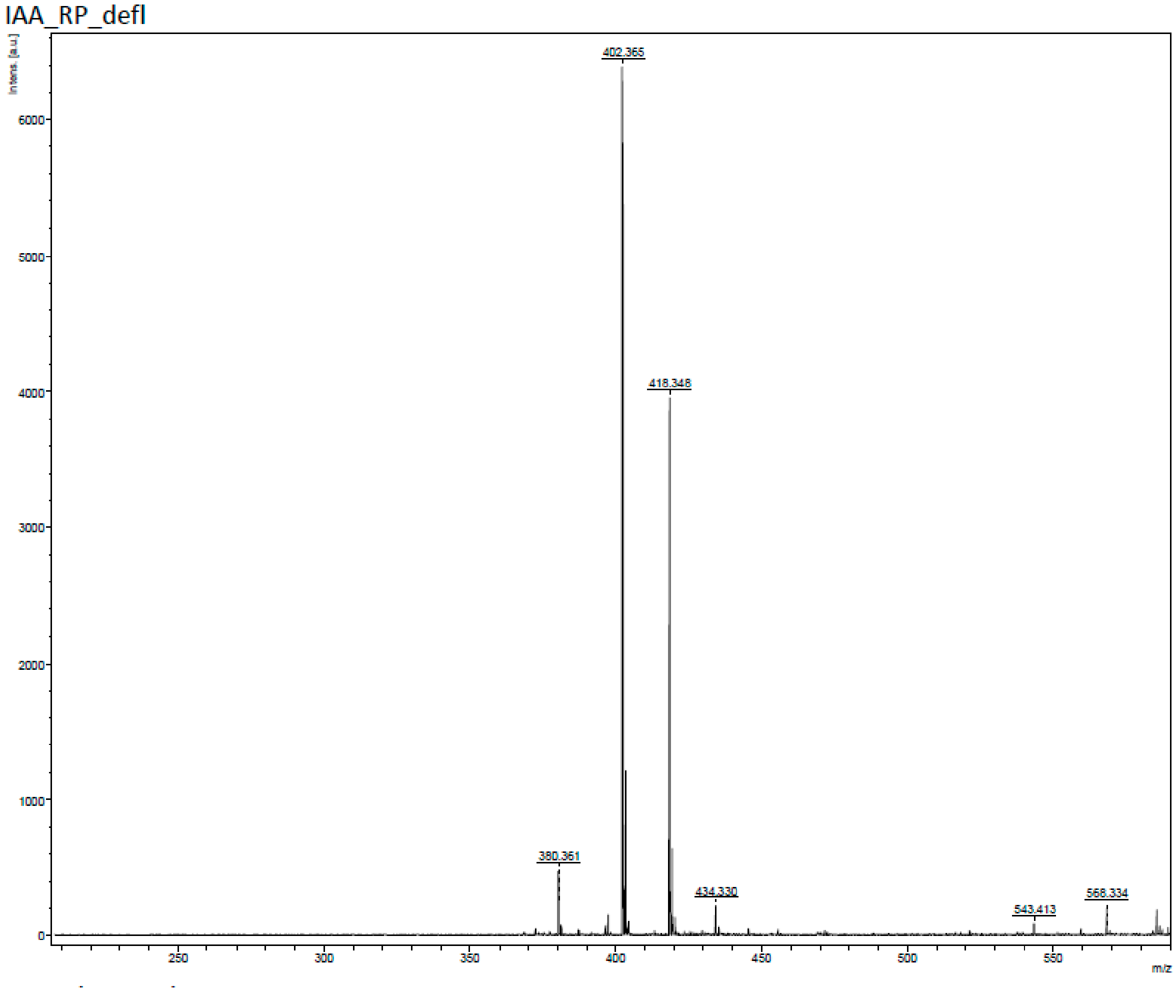

MS (MALDI-TOF): m/z calcd. for $C_{17}H_{18}FN_3O_7$ 395.34; founded 418.35 [M+Na$^+$].

^{1}H NMR of compound **SN_17**

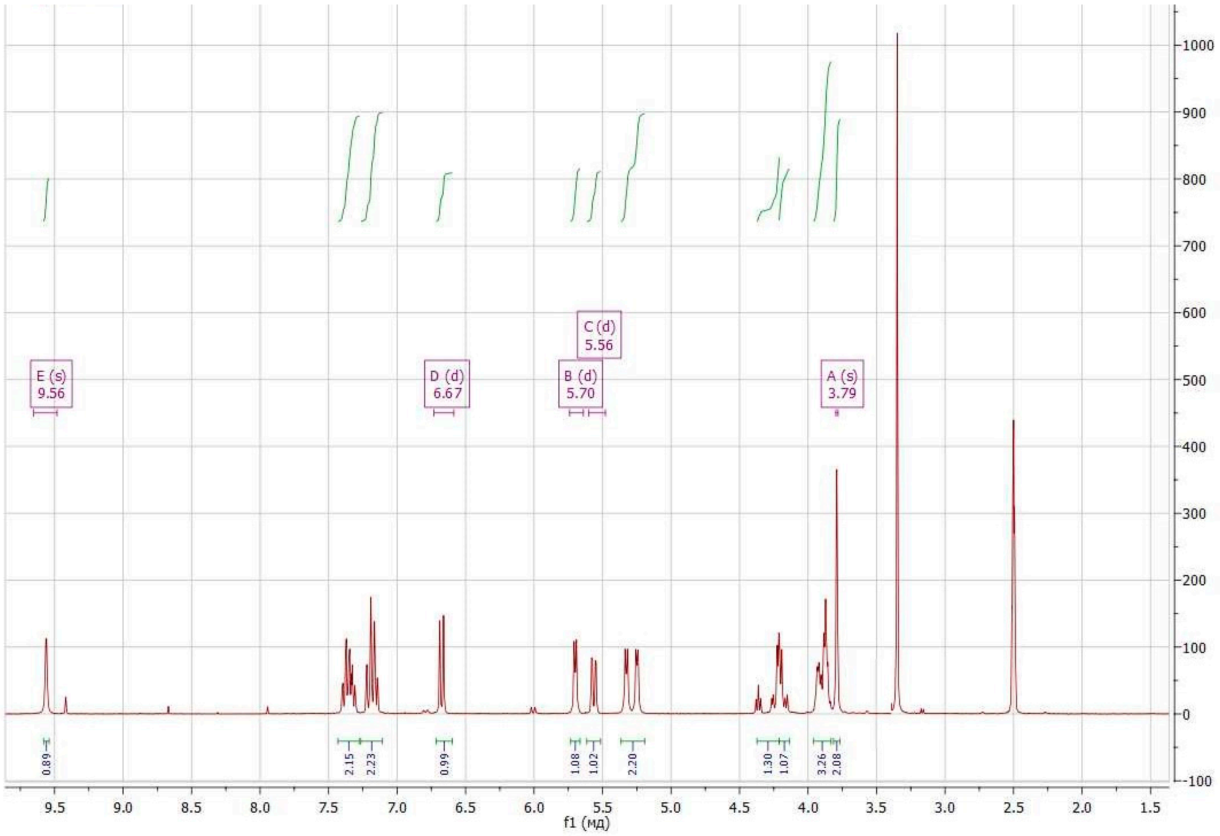

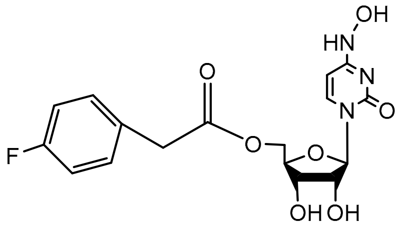

Molecular Weight: 395,34

5'-O-(4-fluorophenyl)acetyl-N4-hydroxycytidine (18, SN_18)

3-Indoleacrylic acid, IAA

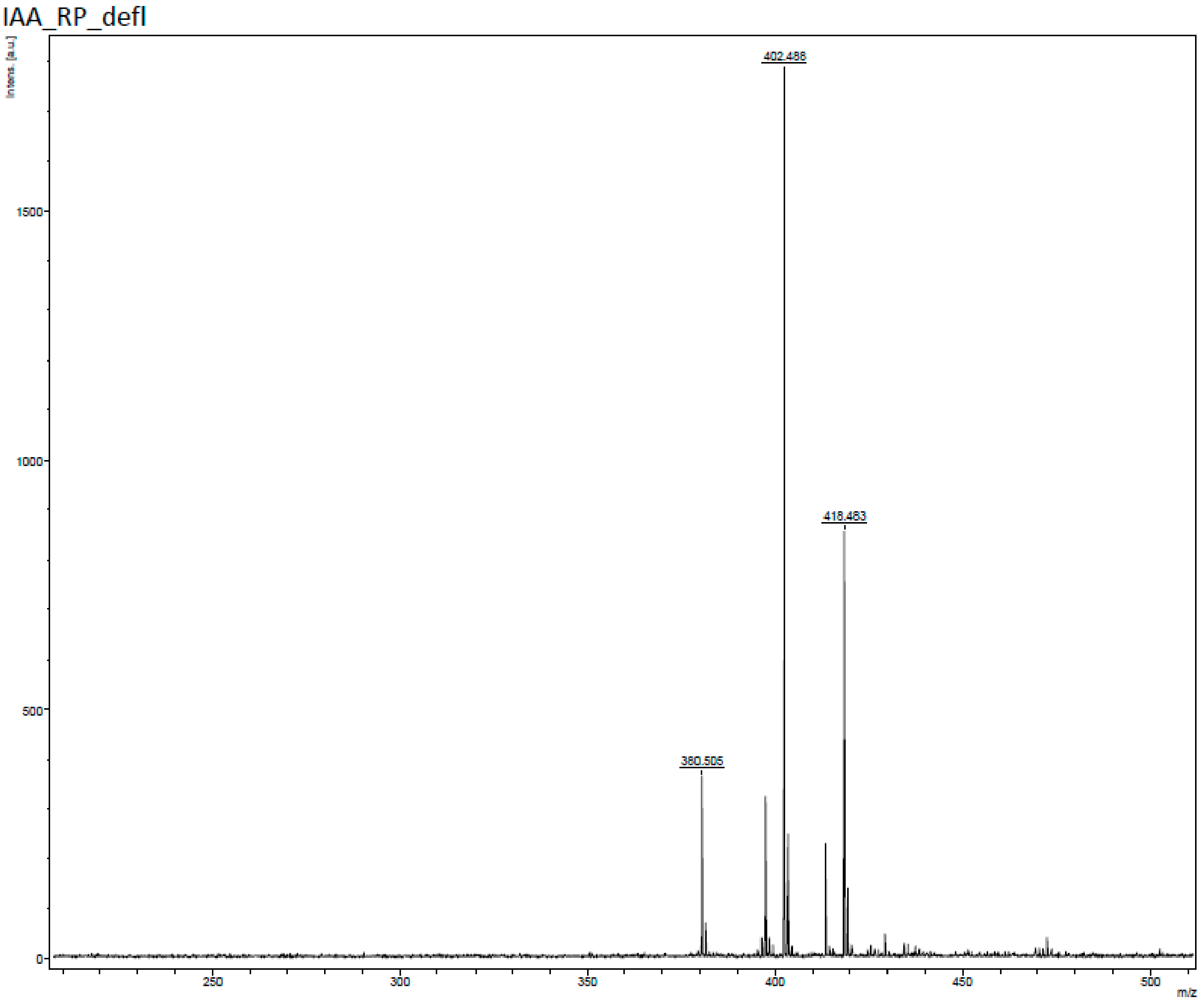

MS (MALDI-TOF): m/z calcd. for $C_{17}H_{18}FN_3O_7$ 395.34; founded 418.48 [M+Na$^+$].

^{1}H NMR of compound **SN_18**

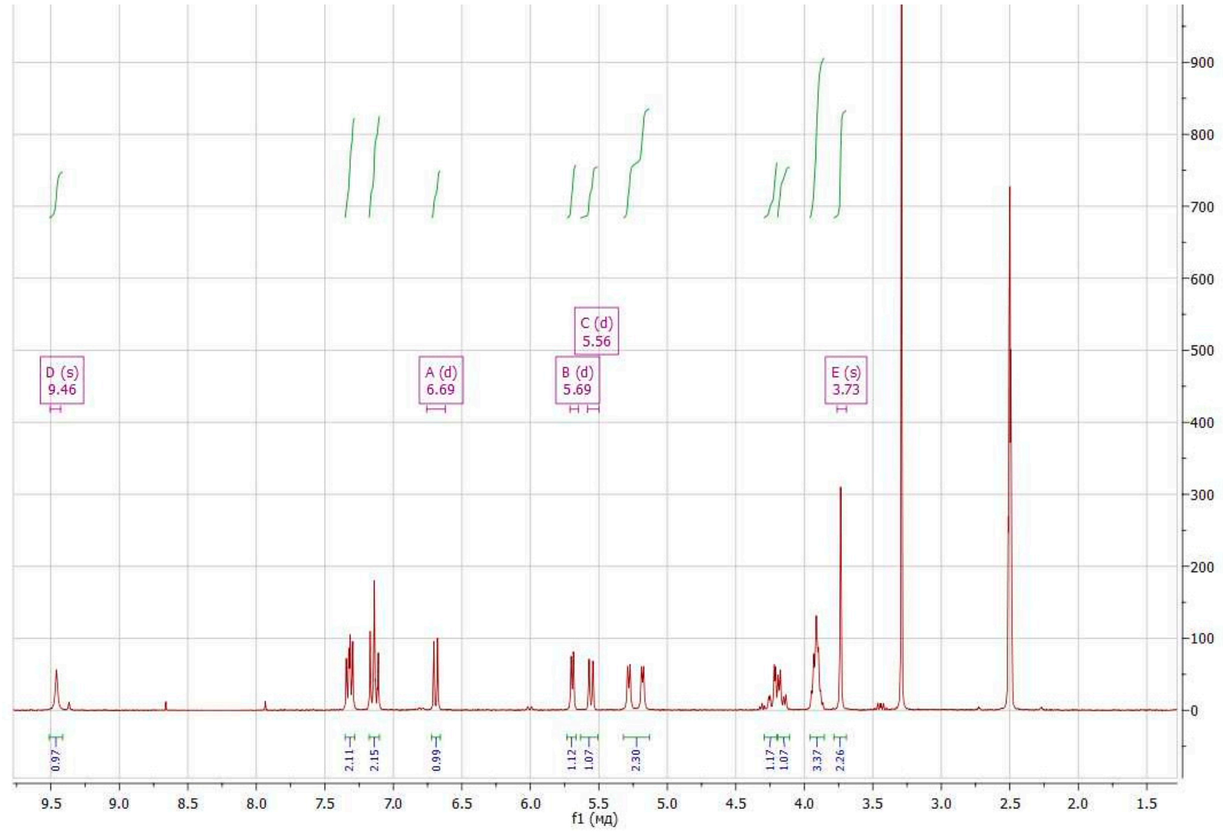

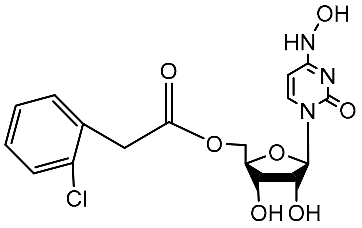

Molecular Weight: 411,80

5'-O-(2-chlorophenyl)acetyl-N4-hydroxycytidine (19, SN_19)

3-Indoleacrylic acid, IAA

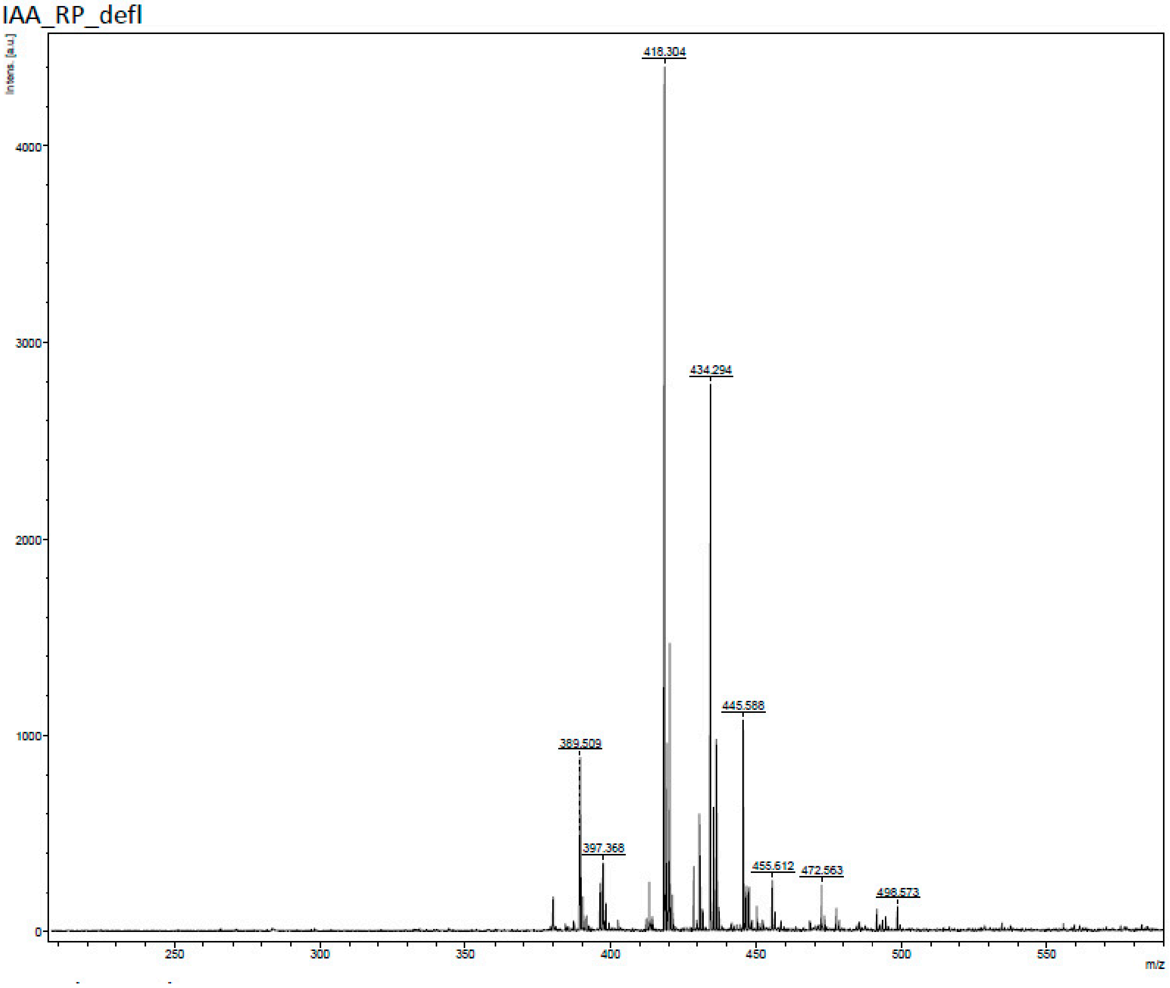

MS (MALDI-TOF): m/z calcd. for $C_{17}H_{18}ClN_3O_7$ 411.80; founded 434.29 [M+Na$^+$].

^{1}H NMR of compound **SN_19**

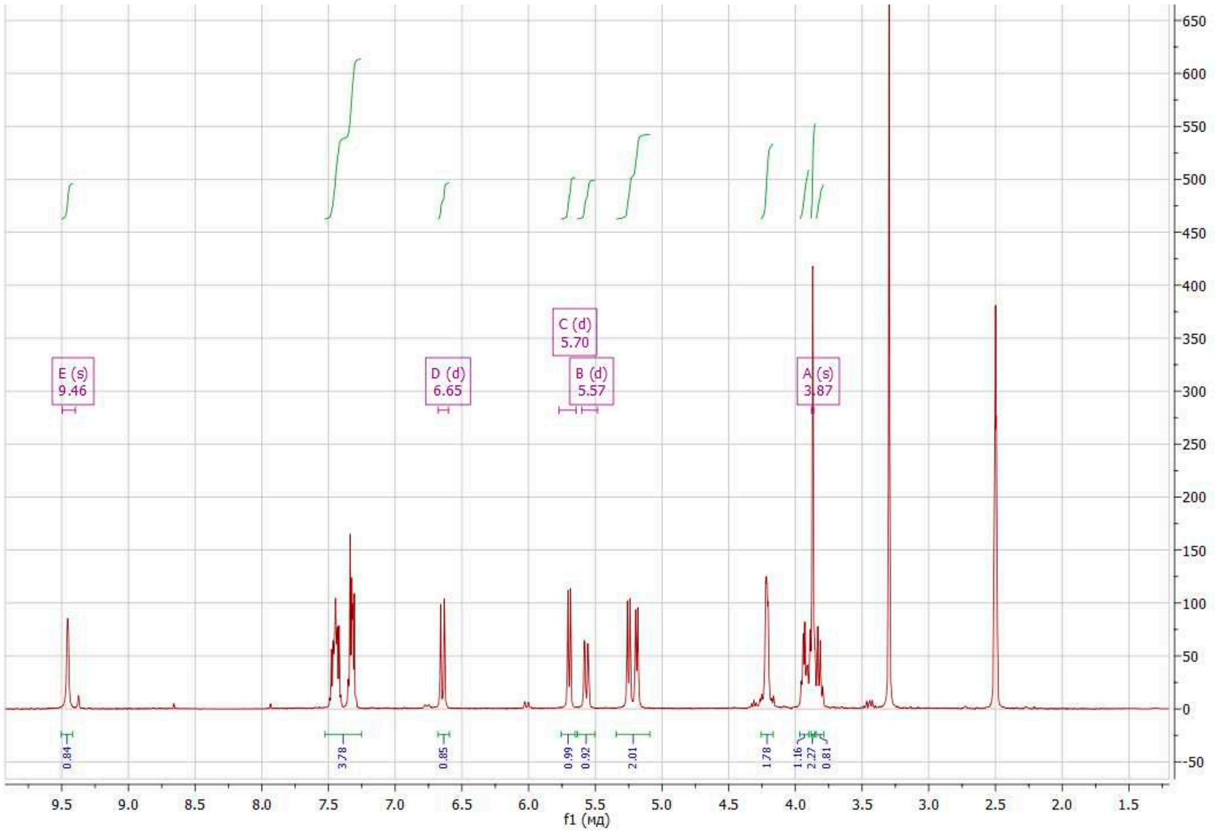

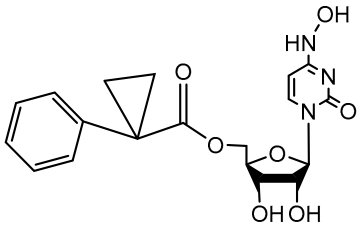

Molecular Weight: 403,39

5'-O-(1-phenylcyclopropanoyl-1-carbonyl)-N4-hydroxycytidine (20, SN_20)

α-Cyano-4-hydroxycinnamic acid, CHCA

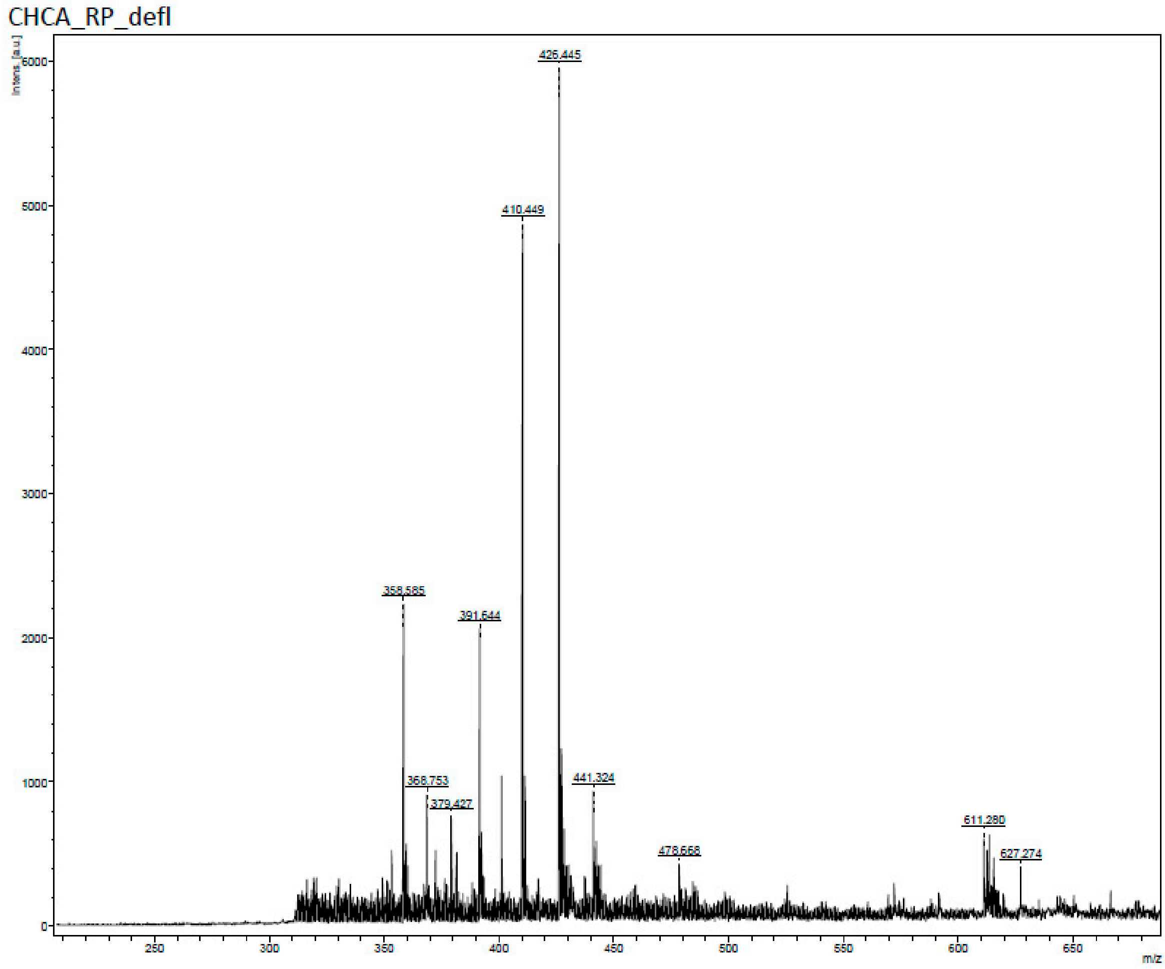

MS (MALDI-TOF): m/z calcd. for $C_{19}H_{21}N_3O_7$ 403.39; founded 426.45 [M+Na$^+$].

^{1}H NMR of compound **SN_20**

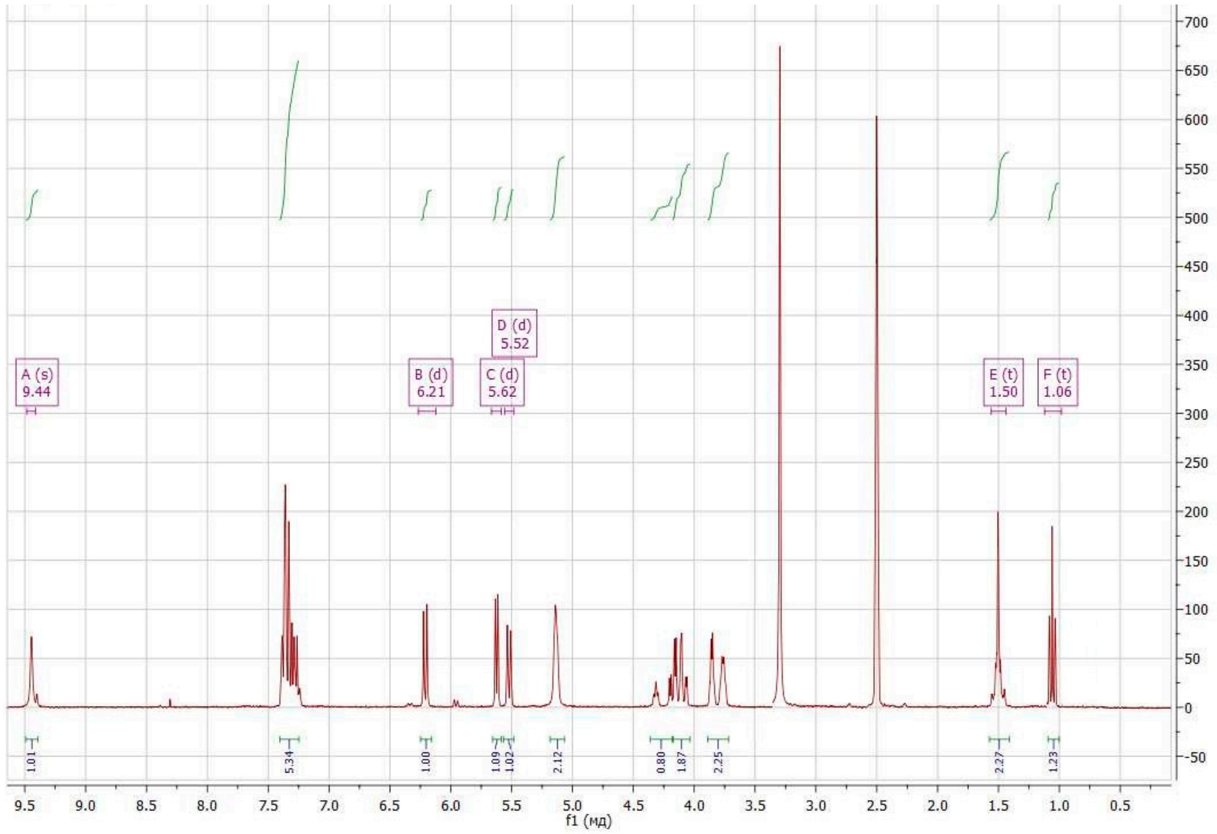

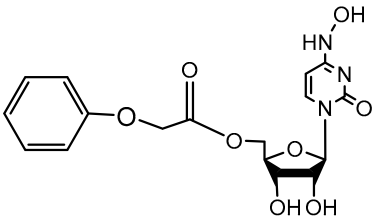

Molecular Weight: 393,35

5'-O-phenoxyacetyl-N4-hydroxycytidine (21, SN_21)

Sinapic Acid

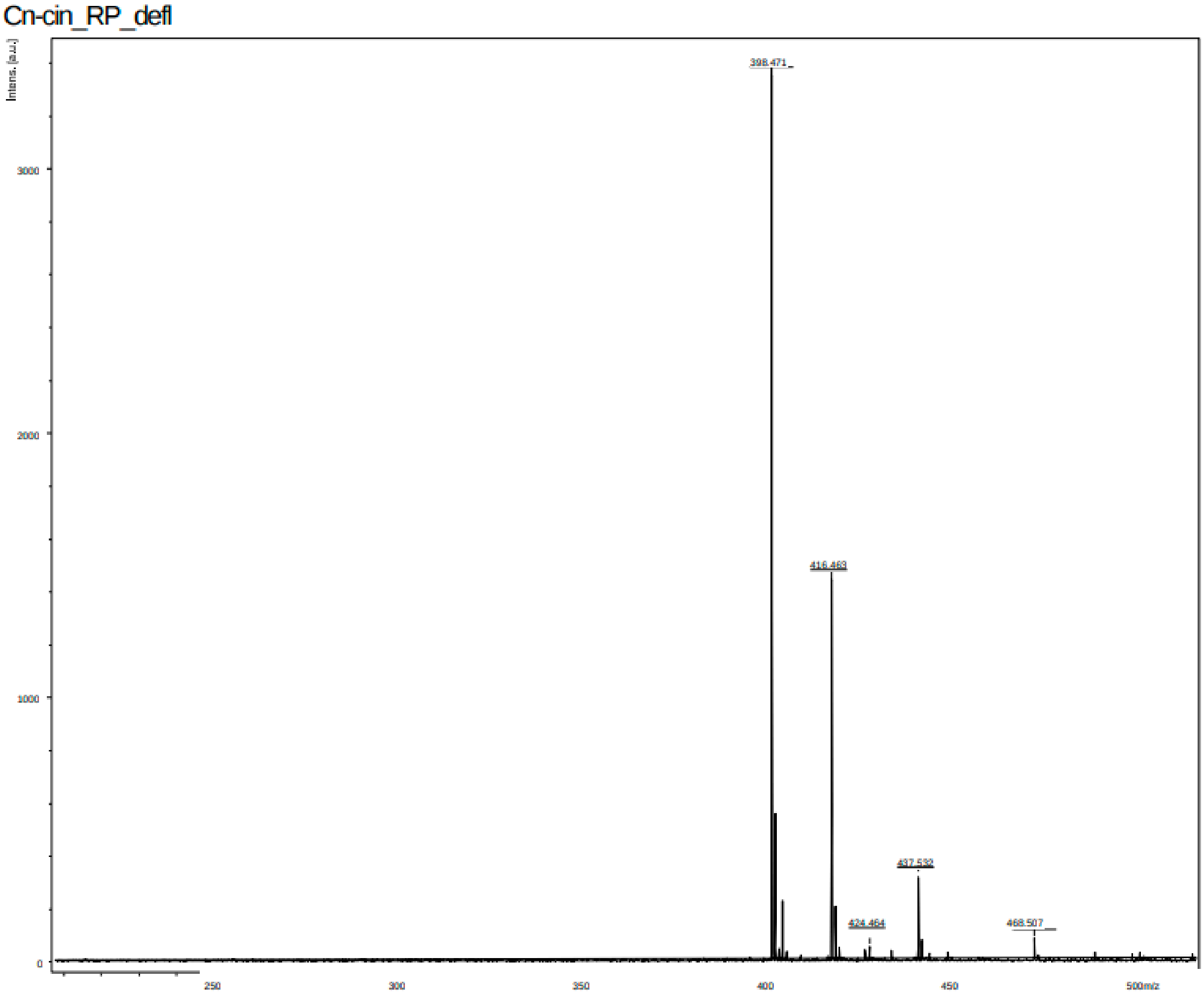

MS (MALDI-TOF): m/z calcd. for $C_{17}H_{19}N_3O_8$ 393.35; founded 416.46 [M+Na$^+$].

^{1}H NMR of compound **SN_21**

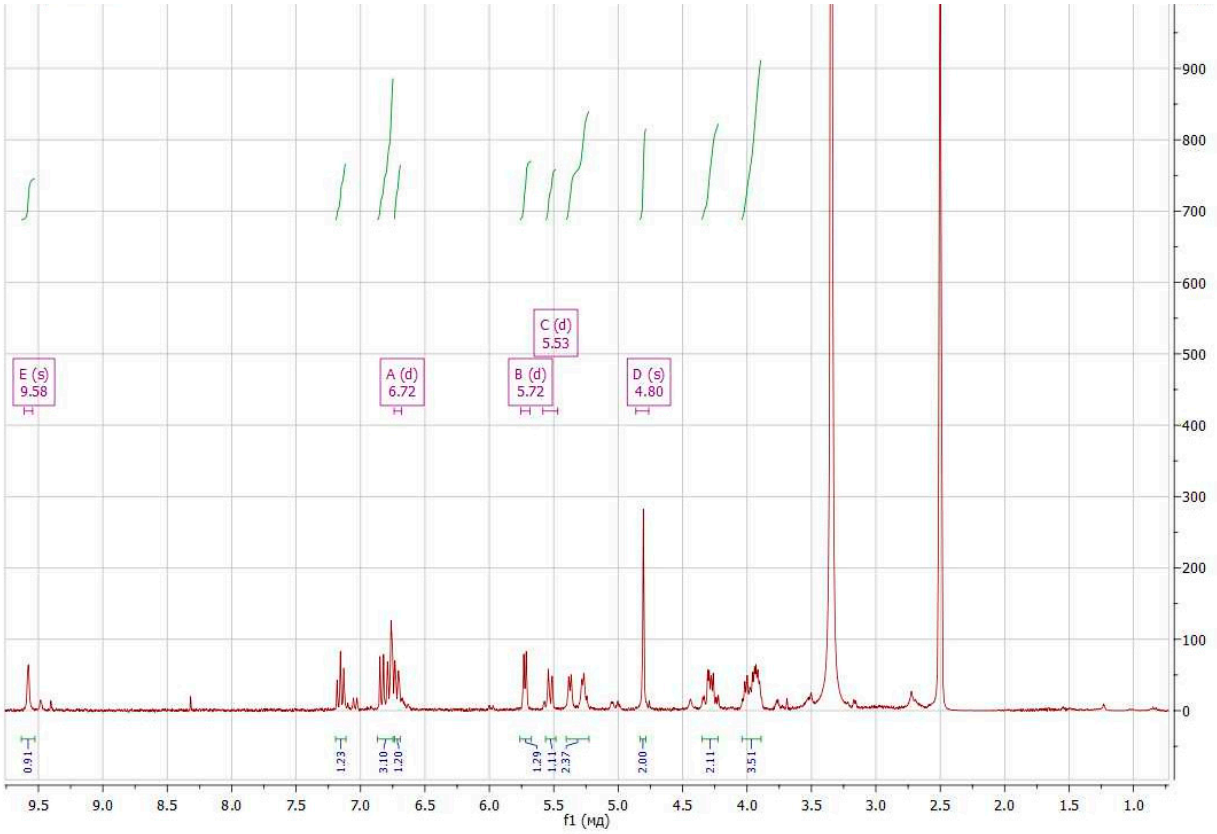

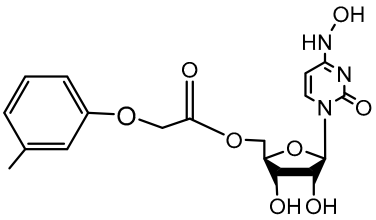

Molecular Weight: 407,38

5'-O-(3-methylphenoxy)acetyl-N4-hydroxycytidine (22, SN_22)

Sinapic Acid

Cn-cin_RP_defl

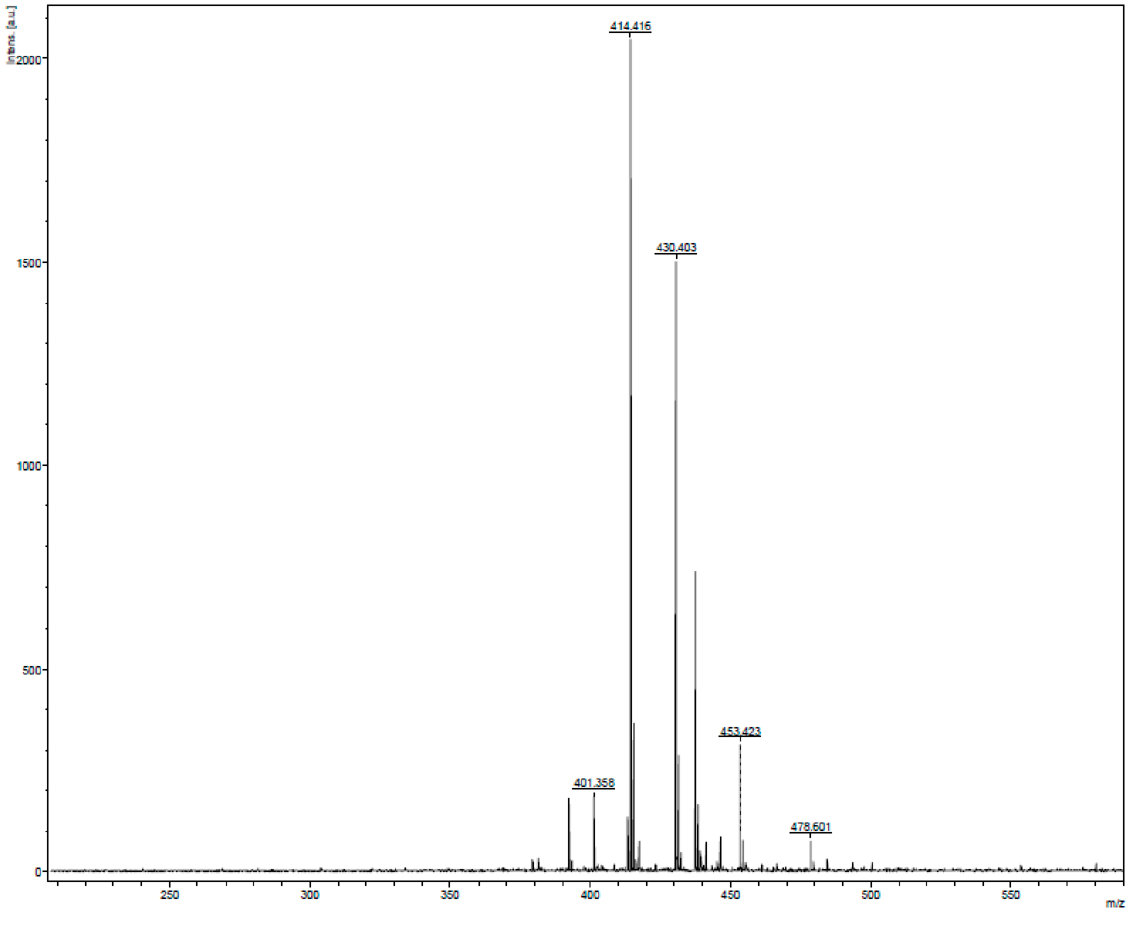

MS (MALDI-TOF): m/z calcd. for $C_{18}H_{21}N_3O_8$ 403.38; founded 430.40 [M+Na$^+$].

^{1}H NMR of compound **SN_22**

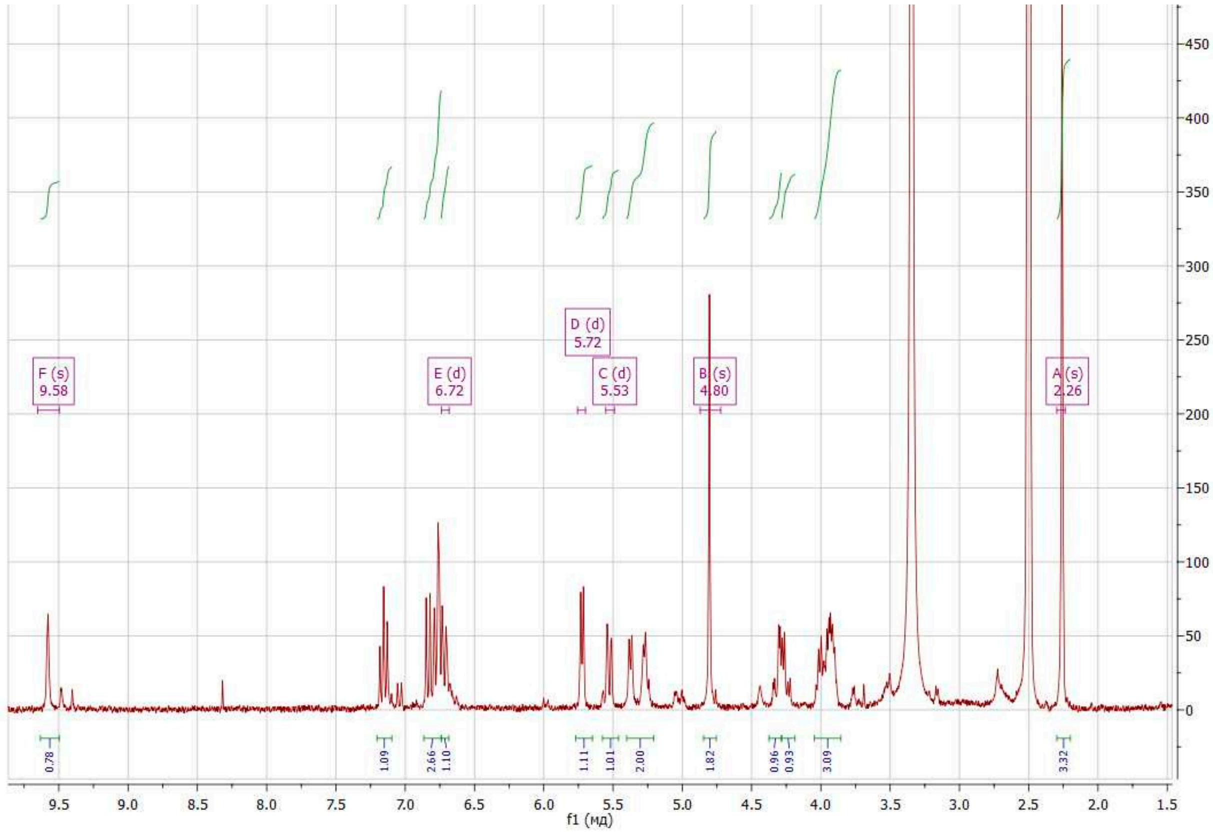

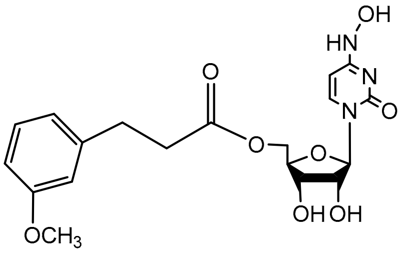

Molecular Weight: 421,41

5'-O-3-(3-methoxyphenyl)propanoyl-N4-hydroxycytidine (23, SN_23)

3-Indoleacrylic acid, IAA

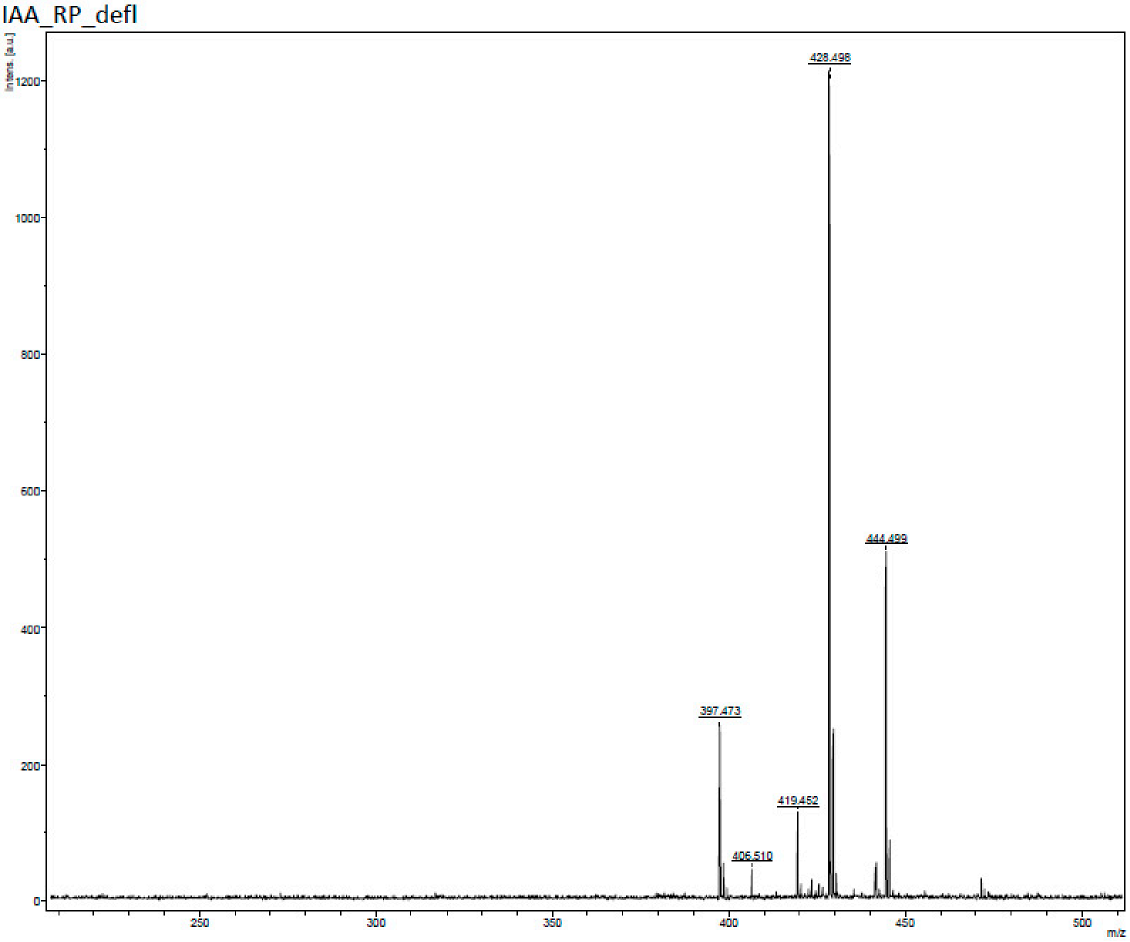

MS (MALDI-TOF): m/z calcd. for $C_{19}H_{23}N_3O_8$ 421.41; founded 444.50 [M+Na$^+$].

^{1}H NMR of compound **SN_23**

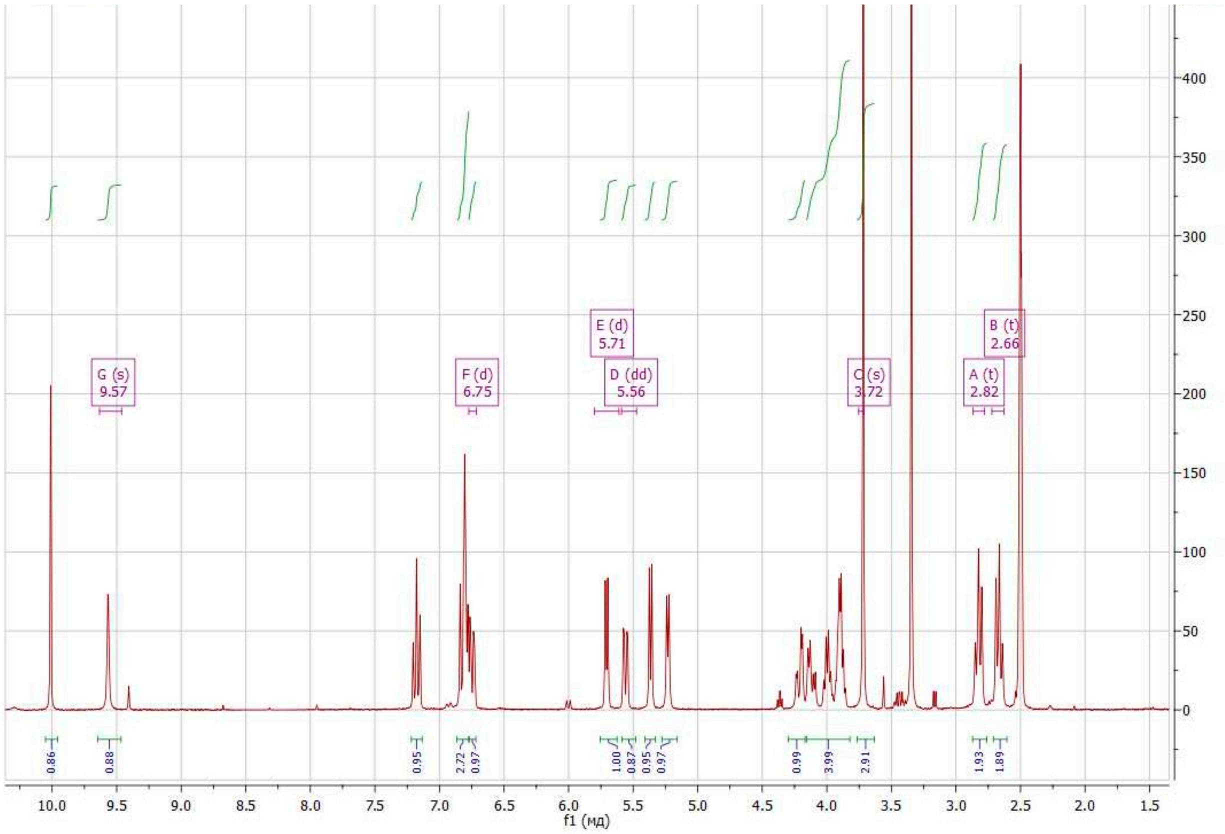

Supplement: Supplementary file 1 [file pharmaceuticals-17-00035-s001.zip › pharmaceuticals-2775518-supplementary.pdf]
